# Amine Hole Scavengers Facilitate Both Electron and Hole Transfer in a Nanocrystal/Molecular Hybrid Photocatalyst

**DOI:** 10.1021/jacs.2c13464

**Published:** 2023-01-27

**Authors:** Sara T. Gebre, Laura M. Kiefer, Facheng Guo, Ke R. Yang, Christopher Miller, Yawei Liu, Clifford P. Kubiak, Victor S. Batista, Tianquan Lian

**Affiliations:** † Department of Chemistry, Emory University, Atlanta, Georgia 30322, United States; ‡ Department of Chemistry and Biochemistry, University of California, San Diego, 9500 Gilman Drive, MC 0358, La Jolla, California 92093, United States; § Department of Chemistry, Yale University, New Haven, Connecticut 06511, United States

## Abstract

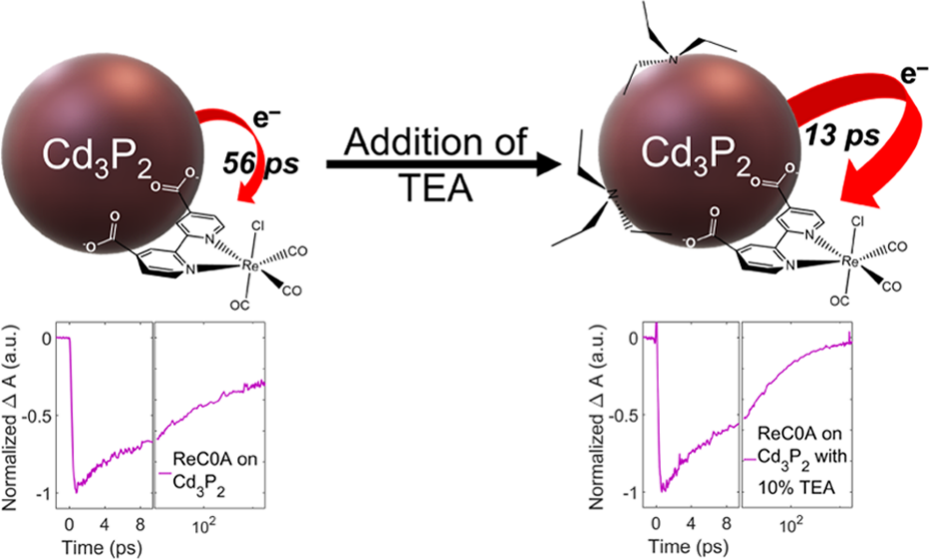

A well-known catalyst, *fac*-Re(4,4′-R_2_-bpy)(CO)_3_Cl (bpy = bipyridine; R = COOH) (ReC0A), has been widely studied for CO_2_ reduction; however, its photocatalytic performance is limited due to its narrow absorption range. Quantum dots (QDs) are efficient light harvesters that offer several advantages, including size tunability and broad absorption in the solar spectrum. Therefore, photoinduced CO_2_ reduction over a broad range of the solar spectrum could be enabled by ReC0A catalysts heterogenized on QDs. Here, we investigate interfacial electron transfer from Cd_3_P_2_ QDs to ReC0A complexes covalently bound on the QD surface, induced by photoexcitation of the QD. We explore the effect of triethylamine, a sacrificial hole scavenger incorporated to replenish the QD with electrons. Through combined transient absorption spectroscopic and computational studies, we demonstrate that electron transfer from Cd_3_P_2_ to ReC0A can be enhanced by a factor of ∼4 upon addition of triethylamine. We hypothesize that the rate enhancement is a result of triethylamine possibly altering the energetics of the Cd_3_P_2_–ReC0A system by interacting with the quantum dot surface, deprotonation of the quantum dot, and preferential solvation, resulting in a shift of the conduction band edge to more negative potentials. We also observe the rate enhancement in other QD–electron acceptor systems. Our findings provide mechanistic insights into hole scavenger–quantum dot interactions and how they may influence photoinduced interfacial electron transfer processes.

## Introduction

Photoreduction of CO_2_ is a promising way to mitigate the environmental impact of CO_2_ and generate solar fuels.^1,2^ Many CO_2_ reduction catalysts are not photoactive or require high-energy photons from the solar spectrum.^3^ A common way of circumventing this issue is utilizing a sensitizer that can absorb light and deliver electrons to the catalyst. Quantum dots (QDs) are excellent sensitizer candidates as they absorb broad ranges of light, their absorption and emission spectra are size tunable, and they can offer stability and also provide a source of electrons.^4−8^ Ideal QD sensitizers must be strongly quantum-confined, have absorption in the visible and near-IR regions, and have a conduction band edge with high enough energy for the catalyst reduction.^9^ Cd_3_P_2_ is such a QD, having a conduction band potential of −1.32 V vs NHE for particles with a band gap of 650 nm.^10^

In this study, we investigate photoinduced electron transfer from Cd_3_P_2_ to the tricarbonyl CO_2_ reduction catalyst Re(4,4′-R_2_-bpy)(CO)_3_Cl (bpy = bipyridine; R = COOH) (ReC0A). Rhenium tricarbonyl catalysts have shown high selectivity and efficiency in reducing CO_2_.^11−16^ However, when used as a photocatalyst, ReC0A exhibits poor absorption of solar light since it requires photons with wavelengths shorter than 400 nm to generate an ^1^MLCT state before it can be reduced. Here, we bind the ReC0A catalyst to Cd_3_P_2_ QDs, so the QDs can both absorb visible light and transfer the photoexcited electrons to the ReC0A adsorbate for catalysis.

A sacrificial electron donor must replenish the QD to sustain the CO_2_ reduction.^1,17^ We have chosen triethylamine (TEA), which is a well-studied hole scavenger. However, like other amines, TEA is known to have complicated effects on quantum dots because it can function as a hole scavenger and as a capping ligand.^18,19^ It has also been shown to function as a passivating ligand to remove hole traps at small concentrations^20^ and shift the conduction band edge to more negative potentials.^21^ In this study, we investigate how the presence of TEA affects photoinduced electron transfer from Cd_3_P_2_ QDs to ReC0A. We show that, while TEA functions as a hole scavenger, it also serves as a base to modify the surface charge of Cd_3_P_2_ QDs, thus increasing the electron transfer rate from the QD to the catalyst.^21,22^

## Results and Discussion

### Sample Preparation and Characterization

**Figure 1:**
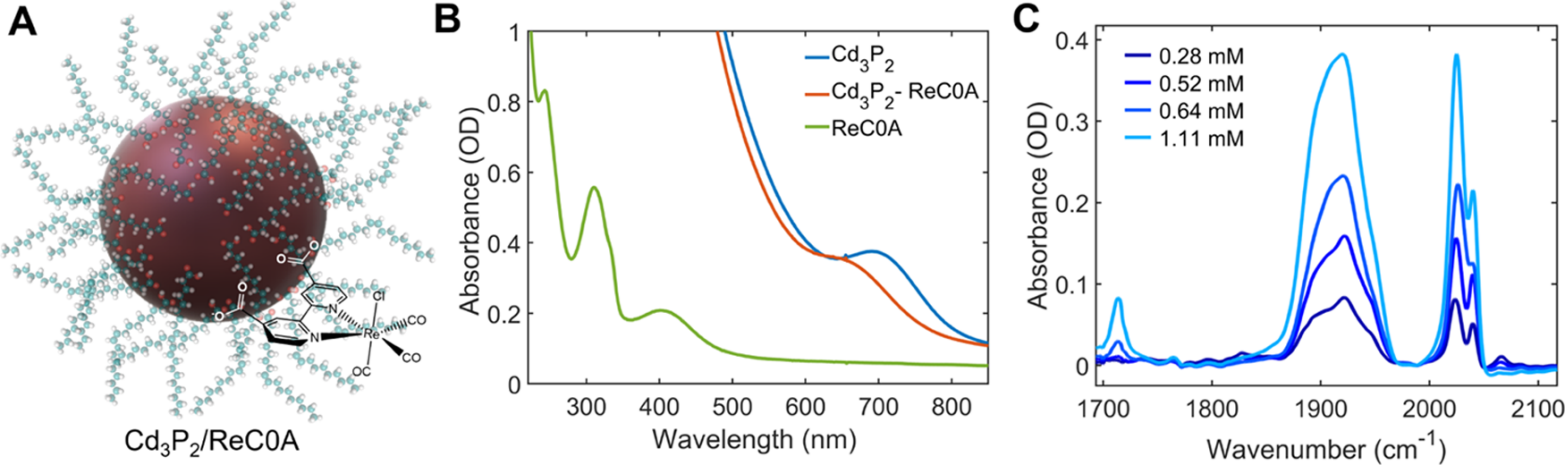
(A) Structure of the catalyst ReC0A. (B) UV–vis spectra of ReC0A in acetonitrile (green), Cd_3_P_2_ in CHCl_3_ (blue), and Cd_3_P_2_-ReC0A in heptane (red). A blue shift occurs when ReC0A is bound to the QD. (C) FTIR of the three carbonyl stretching bands of ReC0A on Cd_3_P_2_, the two high-frequency modes result from coupling due to aggregation. At higher concentrations of ReC0A, protonated COOH groups appear (∼1715 cm^–1^).

Cd_3_P_2_ QDs are particularly suitable for our study since they have a highly reductive conduction band potential and their absorption onset is red-shifted relative to the absorption of the Re catalyst, enabling selective excitation of the QD in the visible range.^10,23^ Recent advances in synthesis methods have enabled the preparation of Cd_3_P_2_ QDs with narrow size distributions under safe conditions.^24−26^ The Cd_3_P_2_ synthesis used in this study is described in detail in Supporting Information 1. Previous studies reported efficient electron transfer from Cd_3_P_2_ QDs to rhodamine B, an electron acceptor with a reduction potential of −0.55 V vs NHE, for Cd_3_P_2_ nanoparticles of band gaps up to ∼1140 nm.^27^ Because the photocatalyst, ReC0A (Figure 1A), has a much more negative reduction potential^28^ (first: −1.06 V vs NHE, second: −1.66 V vs NHE, Figure S18), only QDs with a 1S exciton band shorter than 800 nm are estimated to have a sufficiently high conduction band (CB) edge position to reduce the catalyst. Thus, by incorporating Cd_3_P_2_, the spectral range over which the QD–ReC0A hybrid photocatalyst can be excited extends from ∼400 to ∼800 nm. The QDs in this study show 1S exciton bands at 650–720 nm, which correspond to diameters of ∼2.6–3.1 nm (Figure S1) using a previously published empirical relationship between the QD size and 1S exciton peak position (see Supporting Information 2 for details).^10,23^ Compared to a bulk exciton radius of 18 nm, these QDs are highly quantum-confined.^10,23^ The CB edge electron (1S_e_) and valence band (VB) edge hole (1S_h_) energies for this size Cd_3_P_2_ nanoparticle are estimated to be ∼−1.34 and 0.60 V vs NHE, respectively.^29−31^ The procedure for preparing QD/catalyst complexes in heptane solution is described in Supporting Information 1. The ReC0A catalyst coordinates to the Cd^2+^ sites through the deprotonated carboxylic acid groups,^32^ with either one or two COO^–^ groups bound to the surface depending on the ReC0A concentration. Though each COO^–^ can bind through one or both O atoms, the resulting change in frequency between the two binding motifs is not large enough to be observed.^33^ When ReC0A is bound to the QD, a blue shift of up to 40 nm is observed in the QD 1S exciton band (Figure 1B). One possibility for this drastic blue shift is the etching of the QD surface caused by catalyst binding. The observed blue shift would correspond to a decrease in the QD diameter by 0.5 nm due to the strong quantum confinement. High-resolution transmission electron microscopy (HRTEM) experiments were conducted on Cd_3_P_2_ and ReC0A-bound Cd_3_P_2_ to see if etching was indeed occurring. The images (Figure S2) were analyzed using ImageJ software, and results indicate that the Cd_3_P_2_ (∼720 nm) has a size distribution of 3.1 ± 0.2 nm and the Cd_3_P_2_–ReC0A has a size distribution of 3.3 ± 0.2 nm. We find that the addition of ReC0A does not result in smaller Cd_3_P_2_ QDs. Another possible explanation for the observed blue shift could be that these strongly quantum-confined particles may be sensitive to the change in the surface ligand environment. Replacing the native oleic acid (OA) ligands on the QD surface by ReC0A, which has a large dipole moment (∼8.1 Debye)^34^ and a relatively small HOMO–LUMO gap, may alter the confinement potential and hence the confinement energy.^10^ We did not observe this blue shift when ReC0A was bound to CdS or CdSe QDs (Figure S5), which are both less quantum-confined than Cd_3_P_2_. We attempted to determine if the blue-shifting can be reversed by adding an excess of oleic acid capping ligands (20% by volume) to the QD–ReC0A solution at room temperature, which unfortunately significantly degraded the samples and did not lead to conclusive findings. This experiment was performed again at slightly elevated temperatures. The OA stock was heated to 57 and 108 °C, and upon addition of OA (1–20% by volume) to the QD–ReC0A and QD only samples, there was a continual blue shift of the UV–vis spectrum with increasing amounts of OA added for both samples (Figure S3). It seems that adding an excess amount of OA cannot reverse the observed blue shift. It is likely that OA cannot displace adsorbed ReC0A complexes because they are not soluble in the heptane solution. The most likely reason for the observed shift is the strong interaction of the QD with the adsorbate, forming mixed energy levels.^35^ This effect is strengthened with the addition of more molecules to the QD surface, which is observed with ReC0A (Figure S4). Furthermore, with this effect, the direction of the shift may depend on the relative energy level of the QD and the adsorbate. We have previously observed that the addition of rhodamine B and methyl viologen can result in both blue and red shifts of the Cd_3_P_2_ exciton band, respectively. Although these observations are consistent with this model, it requires further investigation and theoretical modeling, which is ongoing and will no longer be discussed here.

**Figure 2:**
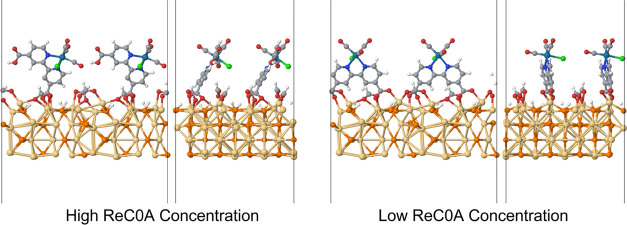
DFT models of the ReC0A catalyst adsorbed on the Cd_3_P_2_ (100) surface at different concentrations. The left panel shows binding modes at high concentrations of ReC0A adsorbed on the surface through a single carboxyl group. The right panel shows that, for low concentrations, ReC0A adsorbed on the surface through both carboxyl groups. The binding of the second carboxyl group is responsible for the disappearance of the 1700 cm^–1^ peak in the experimental absorbance spectrum when lowering the ReC0A concentration (seen in Figure 1).

Our DFT analysis of binding modes provides a first-principles interpretation of the FTIR spectra of ReC0A bound to Cd_3_P_2_ surfaces (Figure 2) as compared to the spectrum of ReC0A in acetonitrile where the catalyst exhibits three carbonyl stretching modes in the 2000 cm^–1^ region corresponding to an out-of-phase symmetric stretch (1905 cm^–1^), an asymmetric stretch (1922 cm^–1^), and an in-phase symmetric stretch (2025 cm^–1^). When ReC0A is bound to Cd_3_P_2_, two bands appear for the symmetric stretch at 2025 and 2040 cm^–1^ (Figure 1C). It was previously reported that this band splitting is the result of coupling of the carbonyl stretches due to aggregation, specifically with two bands in the dimer and three bands in a trimer.^33,36^ This is a reasonable assumption for ReC0A on Cd_3_P_2_, since the average ReC0A per QD is relatively high, at 21 molecules for a concentration of 0.49 mmol ReC0A. Since the Re compound is not soluble in heptane, all complexes observed in the FTIR spectra are assumed to be bound to the QD. With this number of Re molecules on the surface, it is possible that some of the oleic acid (OA) ligands are removed during the adsorption process. Our computational modeling of the interaction between capping oleic acid (OA) ligands and Cd_3_P_2_ suggests that the carboxyl groups of OA are deprotonated and protons prefer to bind to the surface P sites. The same binding motif is observed when ReC0A molecules are adsorbed to Cd_3_P_2_ surfaces. At low concentrations of ReC0A, both carboxyl groups of ReC0A bind to the surface Cd^2+^ site in the deprotonated form. Therefore, no IR peak is observed around 1715 cm^–1^ for ReC0A at concentrations of 0.28 and 0.54 mM. As the concentration of ReC0A increases, due to the limited Cd_3_P_2_ surface area, ReC0A molecules begin to bind to the Cd_3_P_2_ surface through a single carboxyl group, with the other protonated COOH group pointing away from Cd_3_P_2_ QDs, giving rise to the characteristic IR peak at ∼1715 cm^–1^ (Figure 2). According to the energetics of the adsorbate obtained by DFT calculations, the estimated minimum concentration of ReC0A necessary for transition from a bi-carboxylate binding mode to a mono-carboxylate binding motif is 0.7 mM (see Figure S16). This confirms that the appearance of the IR peak at ∼1715 cm^–1^ (at high concentrations of ReC0A) is due to that transition. Binding of carboxyl groups to Cd^2+^ sites is concerted with deprotonation of the carboxylic acid group.

Cyclic voltammetry (CV) experiments were conducted on the ReC0A and ReC0A/TEA systems to determine their respective reduction potentials. In a solution without TEA, the first reduction, which is known to be bipyridine-based,^37^ is at −1.06 V vs NHE. Upon addition of TEA, the first reduction of the ReC0A is pushed more negative to −1.14 V vs NHE and a new, small peak appears at −1.29 V vs NHE (Figure S18). The shift in the first reduction potential is due to the acid–base chemistry between TEA and the bpy-C0A ligand. The new peak corresponds to the reduction of the formed triethylammonium (TEAH^+^). The second reduction of the complex, which is known to be metal-based,^37^ has the same potential (−1.66 V vs NHE) for both the protonated and deprotonated ReC0A species. This is also true for the third reduction at −2.20 V vs NHE. To determine if the origin of the shift of the first reduction and of the new peak at −1.29 V vs NHE resulted from acid–base chemistry of the ligand with TEA, control experiments were conducted using Re(bpy)(CO)_3_Cl (ReCl) (Figure S17). The first reduction of ReCl remained the same (−1.16 vs NHE) regardless of the presence of TEA in solution, and there was no appearance of a peak between the first and second reduction of the complex. TEA did not bind to the Re metal center because of its weaker coordinating ability when compared to the chloride ion.^38^

**Figure 3:**
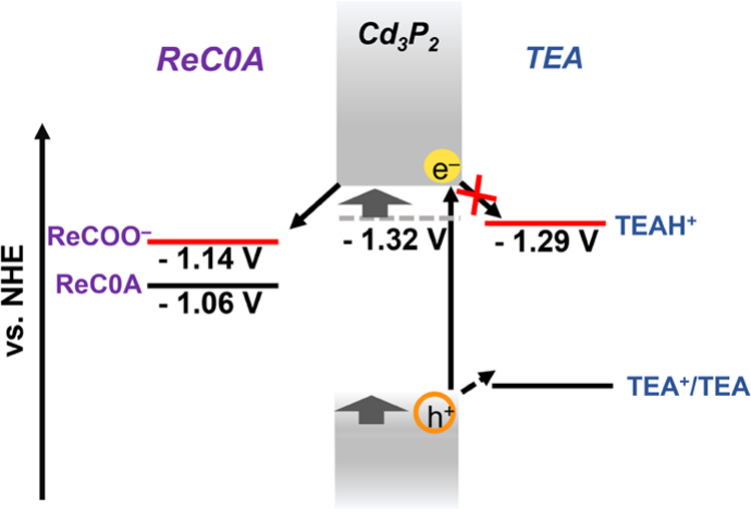
Energy level diagram including energetically allowed electron/hole transfer events. The potentials for ReC0A and TEA come directly from the CV experiments in Figure S18, while the potential for Cd_3_P_2_ comes from the calculation mentioned earlier in this manuscript.

Figure 3 shows the energy level diagram obtained by combining the values determined from the CV experiments, including energetically allowed electron and hole transfer events.

### Addition of Hole Scavenger TEA to QDs

**Figure 4:**
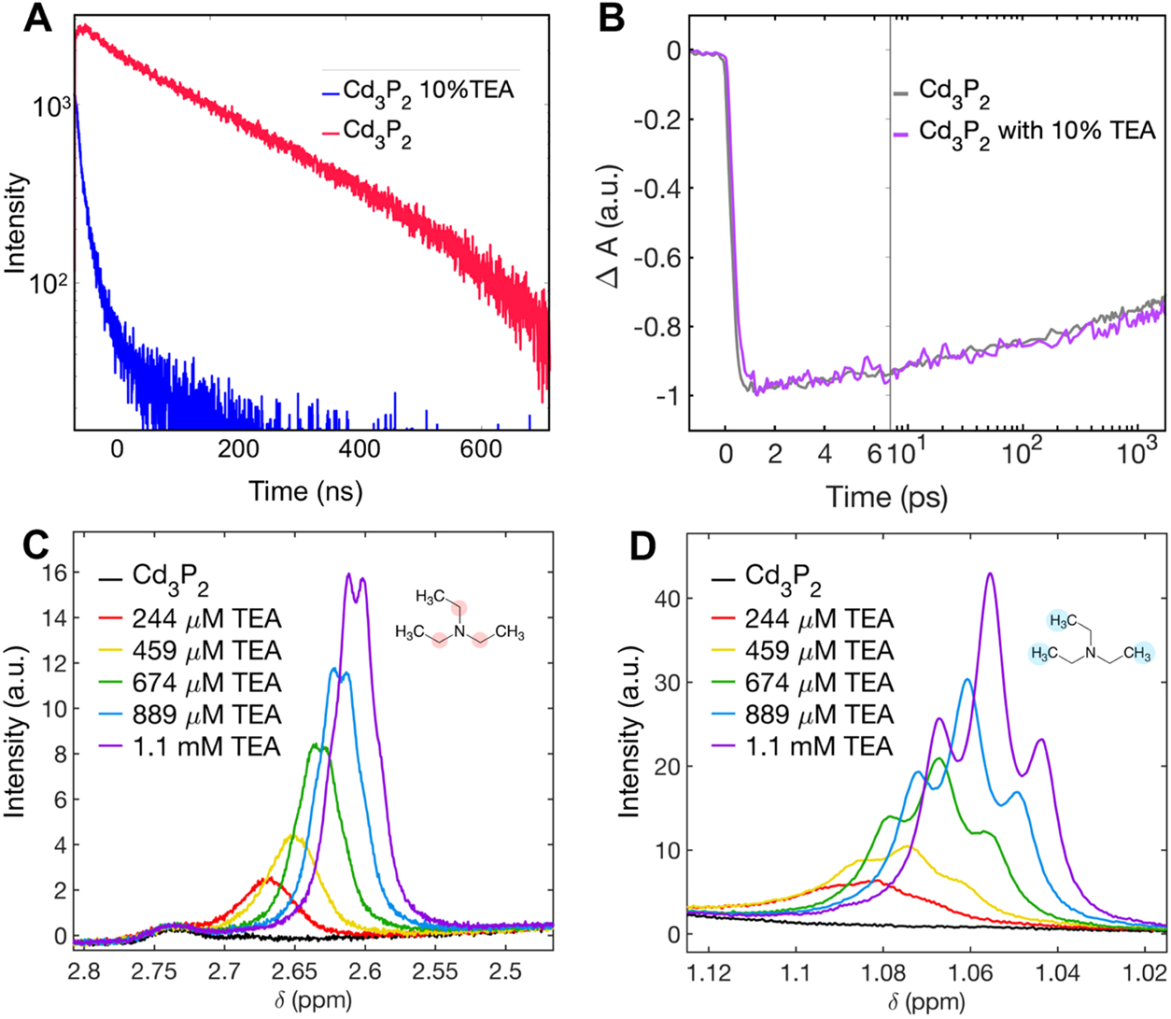
(A) Time-resolved photoluminescence collected at 780 nm showing the decay of Cd_3_P_2_ and how the photoluminescence is quenched by TEA, (B) kinetic traces of 1S exciton bleach of Cd_3_P_2_ with and without 10% TEA, (C) ^1^H NMR spectra of the ethyl (∼2.6 ppm) and (D) methyl (∼1.06 ppm) regions of TEA and how the chemical shifts change as a function of the TEA concentration.

We investigated whether TEA could function as a hole scavenger for Cd_3_P_2_ QDs since Cd_3_P_2_ QDs have not been extensively studied as a photosensitizer. Previous studies using CdSe have reported that adding low concentrations of TEA enhances the QD fluorescence. The enhancement occurs as a result of the hole traps being filled upon binding of TEA.^39^ At higher concentrations, TEA scavenges the holes, causing photoluminescence (PL) quenching.^40,41^ We observed the same effect on Cd_3_P_2_ over a range of concentrations. Time-correlated single-photon counting (TCSPC) experiments with TEA concentrations of 7.1–710 mM (0.1–10% vol) were conducted by exciting the QD with 400 nm light and detecting its PL at 780 nm. These experiments show that, at low concentrations of TEA (up to 35 mM), there is a slight PL enhancement (Figure S13B). The TEA concentration that results in enhancement is much higher with Cd_3_P_2_ than with CdS or Cd_2_As_3_ for which PL enhancement has been reported with up to 1 mM TEA.^42^ At higher concentrations of TEA, the initial amplitude of the Cd_3_P_2_ PL is lower than that of the free QD (Figure S13C). In all cases above 35 mM, TEA quenched the PL (decay rate of 87.6 ± 4.59 ns), confirming that TEA does indeed behave as a hole scavenger in this system (Figures 4A and S6C).

**Figure 5:**
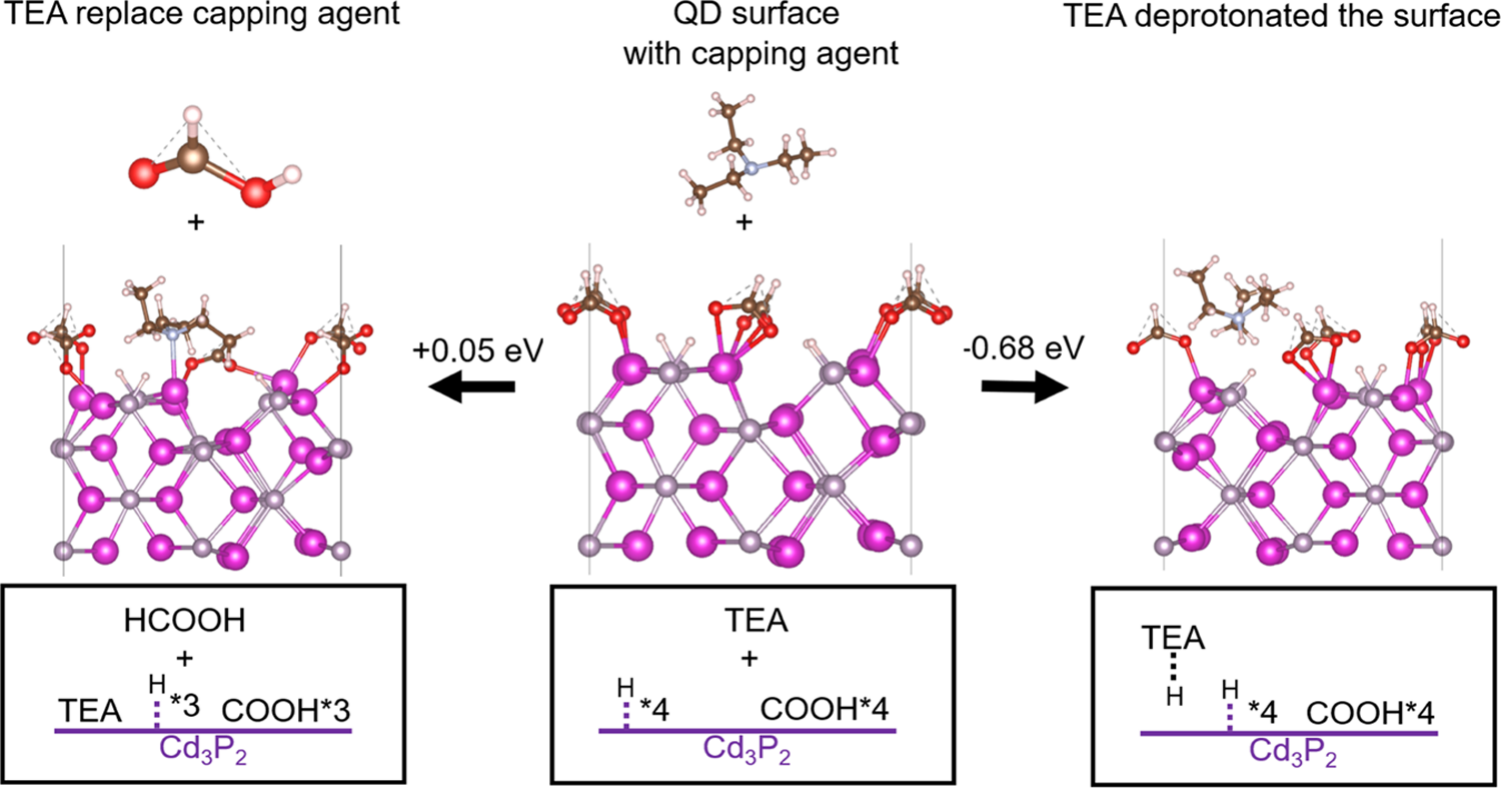
Two possible reaction pathways after adding a TEA molecule: (1) the TEA molecule deprotonates a surface-bound proton and (2) the TEA molecule replaces a capping agent (HCOOH) molecule.

It is difficult to quantify by PL quenching how much TEA is on the QD surface since the two effects on PL intensity occur at varying concentrations of TEA. It is well known that primary amines bind to QD surfaces and they are often used as capping ligands,^19^ but it remains uncertain whether tertiary amines also bind directly to the QD surface. When TEA was introduced for ligand exchange on CdSe, using ^13^P NMR, Scaiano et al. observed the removal of the capping ligand, trioctylphosphine oxide (TOPO), suggesting that TEA binds to the surface.^40^ Our DFT computational analysis suggests that TEA does not replace the capping groups since it is energetically unfavorable. However, the deprotonation of the surface P–H groups by TEA is energetically favorable (Figure 5). We conducted ^1^H NMR titration experiments on the Cd_3_P_2_ in CDCl_3_, adding consecutive amounts of TEA to reach different concentrations to possibly observe a similar loss of the capping ligand (Figure S14A). Surprisingly, we observed a concentration-dependent deshielding of the CH_2_ and CH_3_ protons of the TEA (at 2.6 and 1.06 ppm, respectively), with the highest degree of deshielding occurring at low concentrations, compared to the ^1^H NMR spectrum of free TEA in CDCl_3_ (Figure 4C,D). This suggests that either TEA is preferentially solvating the QD^43^ or that TEA is loosely bound to the QD and exchanges at a rate faster than the experimental resolution without displacing the native OA, consistent with DFT calculations. The former case implies that, at the smallest concentrations of TEA, the environment experiences the largest perturbance (for the smallest concentrations, all TEA are protonated by the surface P–H groups, leading to a larger chemical shift of CH_2_ and CH_3_ protons). As more TEA is added, the environment gradually changes toward that of free TEA and thus becomes more shielded in the NMR experiments. In the latter case, as more TEA is added, while fast exchange still occurs, there is a higher fraction of unbound species, shifting the average to favor the direction of free TEA NMR peaks. The NMR experiments did not determine whether the protons are being extracted from the surface, possibly due to the high concentration of TEA compared to the QD capping ligands (surface P–H groups as a result of surface ligand deprotonation are likely to be the proton source according to our computational modeling) and the limited sensitivity of the instrument, though it is known that TEA is routinely used for surface proton removal in QD synthesis.^22^ While preferential solvation and proton abstraction are most likely occurring, we also observe changes in the OA peaks upon addition of TEA. At the lowest concentrations of added TEA (244 and 459 μM), we observe broadening on the more deshielded side of the OA bands (5.3 and 1.25 ppm for the CH and CH_3_ groups respectively, Figure S14B,C) that recover when more TEA is added. This suggests that, when there is little TEA present, it seeks out the QDs and deprotonates the surface P–H groups, positioning itself near the hydrophilic surface rather than being in the nonpolar heptane environment. As more TEA is added, the protonated TEA form (TEAH^+^) is better solvated by the excess TEA molecule; therefore, it enters the bulk solution and leaves the QD surface, explaining the disappearance of the broadening of the OA peaks. It could also mean that preferential solvation occurs at low concentrations of TEA, as broadening is an indication of a different solvation environment,^44^ and the broadening occurs only at lower TEA concentrations. There is not sufficient evidence that there is strongly bound TEA since there is no permanent peak assigned for such a species.

NMR studies based on diffusion ordered spectroscopy (DOSY) were performed to further analyze the nature of the interaction between TEA and QD in these systems. DOSY NMR determines the diffusion coefficients of compounds in solution, allowing for a clearer identification of species in solution than possible with proton or carbon NMR (details in the Supporting Information). Comparing Cd_3_P_2_ with and without TEA, the diffusion coefficients of oleate in each sample were similar (1.10 μm^2^/s vs 1.14 μm^2^/s with TEA), indicating that no oleate ligands were displaced from the surface (Figure S15). In the case of TEA, DOSY was performed on TEA by itself and with the QD, where a difference in coefficients (3.82 μm^2^/s vs 2.94 μm^2^/s respectively), suggesting that some of the hole scavenger may be closely associated or bound to the surface at a low concentration of 0.49 mM (Figure S16). We are not able to explicitly distinguish between bound and free TEA since only one peak is observed. Therefore, it is important to note that the smaller diffusion coefficient we observe upon addition of TEA to the QD represents both free and bound TEA populations undergoing fast chemical exchange.

Before attempting to observe electron transfer with ReC0A, transient absorption experiments were performed to confirm that, in the case of TEA, the hole has a negligible contribution to the exciton bleach kinetics. Using a mixture of Cd_3_P_2_ in a 10% TEA/90% heptane solution, no difference was observed between the exciton bleach kinetics of the mixture and that of the free QD within 1 ns, indicating that there is a negligible hole contribution to the 1S exciton bleach (XB) signal (Figure 4B), consistent with a previous report.^10^ Furthermore, we conclude that electron transfer to the TEAH^+^ species has not been observed since electron transfer would result in a faster 1S exciton bleach recovery. Although this process is energetically favorable (Figure 3), the interaction of TEAH^+^ species with the QD surface is likely too weak to enable electron transfer. As shown in Figure 5, computation modeling indicates that, upon deprotonation, the new TEAH^+^ species diffuses away from the surface and into the bulk solution, consistent with our hypothesis from ^1^H NMR studies.

### Electron Transfer in Cd_3_P_2_/ReC0A Complexes

**Figure 6:**
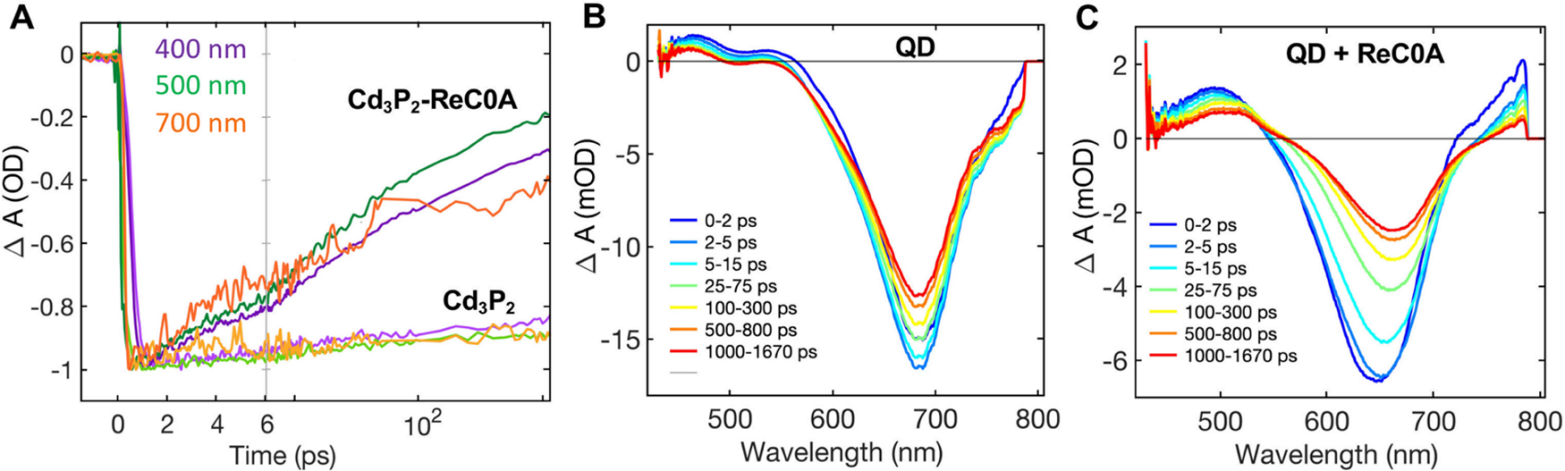
(A) Kinetic traces of the 1S exciton bleach of the QD with and without ReC0A attached. The traces show similar kinetics for excitation wavelengths of 700 nm (orange), 500 nm (green), and 400 nm (purple); TA spectra at multiple time delays of (B) Cd_3_P_2_ and (C) ReC0A bound to Cd_3_P_2_.

Visible transient absorption (TA) spectra of QD–ReC0A complexes were first measured with wavelengths less than the energy needed to electronically excite ReC0A (500 and 700 nm) to establish that electron transfer from the QD to the complex occurs (Figure 6A). All excitation wavelengths resulted in similar QD 1S exciton bleach kinetics. We elected to use 400 nm for the majority of the experiments because it does not interfere with our QD bleach kinetics measurements. Although the estimated concentration of QD is 21 times smaller than that of ReC0A (25 μM vs 525 μM), its molar extinction coefficient of ε_400 nm_ = 530 477 L mol^–1^ cm^–1^ at 400 nm (ε_650 nm_ = 124 700 L mol^–1^ cm^–1^ at the band gap) is 156 times larger than that of ReC0A (ε_400 nm_ ∼3400 L mol^–1^ cm^–1)^.^10,45^ As a result, ReC0A is expected to absorb a negligible amount of light due to the much larger absorptance of the QD. The TA spectra of free QDs (Figure 6B) show a bleach of QD 1S exciton band (XB) centered at ∼680 nm and a photoinduced absorption band at a longer wavelength. It has been shown previously that the XB in Cd_3_P_2_ QDs is dominated by the state filling signal of the 1S_e_ level and can be used to follow the kinetics of the CB edge electron.^10^ The TA spectra of QD–ReC0A complexes (Figure 6C) show similar TA spectra features as free QDs at an early delay time, but the decay of the XB signal is much faster, indicating shorter lived CB electrons due to electron transfer to the adsorbed ReC0A. This is consistent with the observation of TA spectra at ∼520 nm, just over the Cd_3_P_2_ photoinduced absorption (Figure 6C), which can be assigned to the reduced ReC0A species (Figure S6B).^46^ However, the signal at ∼520 nm is overshadowed by the continuously growing charge-separated state signal (electron in ReC0A, hole in VB) which also red-shifts as it grows in.^47^ In the charge-separated state, the exciton transition energy changes due to its interaction with the separated charge, giving rise to a derivative feature with shifted exciton bleach in the TA spectra, which has been observed previously.^48−51^ Additional spectra of Cd_3_P_2_ with methyl viologen excited at 400 nm confirms that this red-shifting signal corresponds to the growth of the charge-separated state (Figure S7). While it is also possible that this signal may correspond to the QDs in solution that have the largest amount of catalysts on the surface (due to the blue shift of the UV–vis spectrum) and therefore contributes to the faster bleach recovery at a higher energy, the growth of the CSS is likely the main cause of the red shift. Both effects can contribute to fast bleach recovery. To corroborate the growth of the reduced ReC0A species in the TA spectra, a separate experiment where a mixture of ReC0A and triethanolamine (TEOA) in MeCN was pumped at 400 nm to generate the excited MLCT and singly reduce the complex was conducted (Figure S6B). As a result, at around 1 μs, once the MLCT state has decayed away, there is a small peak at ∼505 nm corresponding to the reduced ReC0A. Control experiments without TEOA are shown in Figure S6A.

There is an initial loss of an XB amplitude with Cd_3_P_2_–ReC0A, compared to Cd_3_P_2_, on a timescale faster than the instrument response, which is ∼250 fs. This loss can be seen comparing the peak depths of Figure 6B,C, where the ReC0A-bound QD has a depth of just under half of that of the free QD, where both have the same concentrations of QDs. This fast amplitude loss can be caused by either ultrafast electron transfer or electron trapping on defect sites introduced by the adsorption of ReC0A. With regards to the ultrafast ET, comparing the bleach amplitudes of the QD and QD–ReC0A samples upon excitation at 400, 560, and 700 nm will provide insight into whether this process occurs. Figure S10 shows unnormalized kinetics for each sample at each excitation wavelength. Pumping at 400 and 560 nm shows the clearest difference in the bleach amplitude, where 50–60% of the amplitude has disappeared. At 700 nm excitation, the bleach amplitude loss is less, about 30%. Both 400 and 560 nm pumps are at a higher energy than the QD CB edge, so ultrafast ET is more likely to occur, whereas at 700 nm, there is a more direct excitation to the CB edge. However, since there is still bleach amplitude loss for each wavelength, it is likely that the significant cause is due to electron trapping with a possible small degree of ultrafast ET. Upon catalyst adsorption, if ReC0A replaces native OA due to the large volume of ReC0A binding, it will generate surface defects, which can act as electron traps. Both these possibilities contribute to the loss in bleach amplitude, with electron trapping being the main cause. We will further examine this phenomenon in future studies with rhodamine B and will not further discuss it here. Transient IR spectra of the CO stretching mode shows a large positive feature that is consistent with the formation of Fano resonance (FR) signals (Figure S8).^52^ These TRIR experiments were done with Cd_3_P_2_ and varying amounts of ReC0A (0.25×, 1×, and 2× Re) in hexanes under 500 nm excitation at a high pump fluence to investigate. At this experiment’s smallest concentration, 0.25× Re, the FR, a large positive peak, can be observed at ∼2025 cm^–1^. Upon the addition of more ReC0A (1× and 2× Re), the FR signal decays faster than the 0.25× Re case. Also, there is a growth of the singly reduced species peak at ∼2005 cm^–1^ and a ground state bleach at ∼2029 cm^–1^. As more ReC0A is added (1× vs 2×), the amplitudes of both the bleach and singly reduced peak become larger (Figure S8D) and there is a faster decay of the QD electron and FR signals. Both these TRIR and TA experiments demonstrate that Cd_3_P_2_ is indeed able to reduce the catalyst.

**Figure 7:**
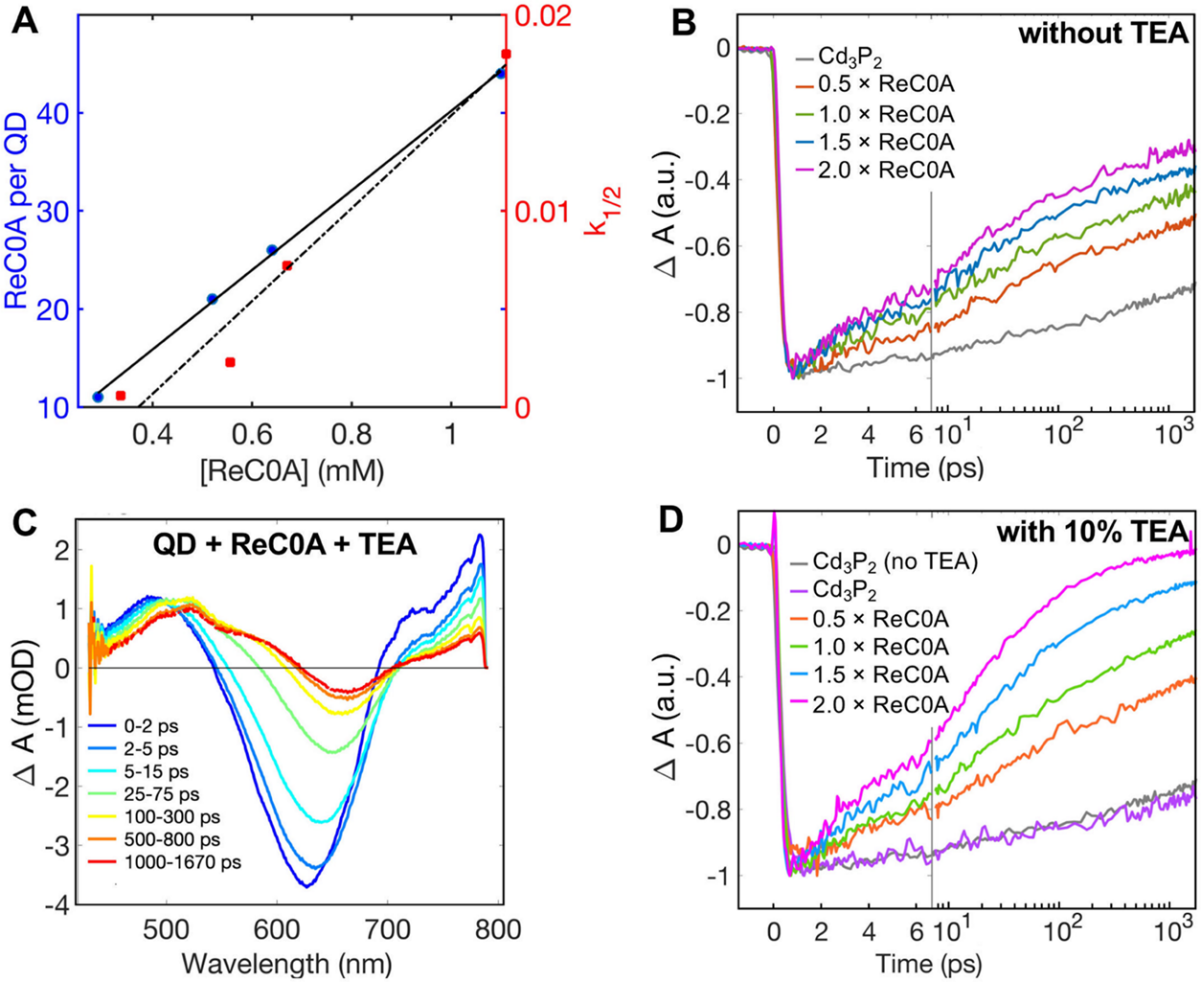
(A) Half-lives of the bleach decays of ReC0A on Cd_3_P_2_ and amount of ReC0A per QD plotted vs ReC0A concentration clearly show a linear relationship. (C) TA spectra at multiple time delays of ReC0A bound to Cd_3_P_2_ with the addition of 10% TEA. The right hand panels show the kinetics of the 1S exciton bleach of Cd_3_P_2_ with multiple concentrations of ReC0A (B) without TEA and (D) with 10% TEA. The addition of TEA shows an enhanced electron transfer rate from Cd_3_P_2_ to ReC0A.

A concentration dependence study was conducted to determine how the electron transfer would be affected as the number of ReC0A molecules on the surface increased. We first observed that, as the concentration increases, so does the blue shift of the 1S exciton band (Figure S11), as well as a greater loss of initial bleach amplitude. Some of the increased loss of the bleach is attributed to scattering as there is an average of 44 ReC0A on the surface for the highest concentration, making Cd_3_P_2_ more susceptible to aggregation in heptane. The concentration study revealed that, as the concentration increased linearly, the bleach recovery rate constant also increased linearly (Figure 7A) with concentrations of 0.28, 0.52, 0.64, and 1.1 mM having half-lives of 1.74 ns, 438.6 ps, 138.5 ps, and 55.6 ps, respectively (Table 1). The average numbers of ReC0A complexes per QD are 11, 21, 26, and 44, respectively, and are thought to adsorb according to a Poisson distribution, as observed. The FTIR spectra (Figure 1C) show that, at the two lowest concentrations of ReC0A on the QD, there is no band at ∼1700 cm^–1^, indicating that all of the carboxylic acids are deprotonated.^53^ As the concentration is increased, a band at 1715 cm^–1^ appears and grows as a function of the ReC0A concentration (Figure 1C, far left). After the surface can no longer accommodate the ReC0As, the ones that are either partially bound (mono-carboxylate) or not bound at all are drawn to the ReC0As on the surface due to their large dipole moment and lack of solubility in heptane. This possible unbound ReC0A environment is drastically different from those of the oleic acids and may affect the reorganization energy for interfacial electron transfer.

**Table 1:** Exciton Bleach Recovery Half-Lives in ReC0A-Cd_3_P_2_ with and without TEA

	ReC0A-QD	ReC0A-QD + 10% TEA
[ReC0A] (mM)	τ_1/2_, no TEA (ps)	τ_1/2_, TEA (ps)
0.28	1740.0 ± 11.9	348.0 ± 16.5
0.52	439.0 ± 12.6	73.5 ± 8.0
0.64	139.0 ± 9.3	28.5 ± 6.4
1.1	55.6 ± 12.1	12.5 ± 0.4

### Effect of TEA on ET in ReC0A/Cd_3_P_2_

Upon addition of TEA to the Cd_3_P_2_–ReC0A system, an increase in electron transfer rate was observed. TEA (10% by volume) was added to each concentration of ReC0A on the QD, and the rate of exciton bleach recovery half-time decreased (Table 1), indicating fast ET rates from the QD to ReC0A. The 1S exciton bleach recovery half-lives for the ReC0A concentrations of 0.28, 0.52, 0.64, and 1.10 mM after adding TEA are 348, 73.5, 28.5, and 12.5 ps, respectively. We observe the presence of a larger reduced ReC0A peak in the TA spectra (∼520 nm) for the experiment containing TEA, indicating that more reduced ReC0A is formed over the same experimental time, consistent with a faster electron transfer to the ReC0A in the presence of TEA (Figures 7B–D and S11). TRIR data show that the amplitudes of reduced ReC0A and the ground state bleach increase with increasing concentrations of ReC0A, providing further evidence for this trend (Figure S8D). Figure S9A,B compares the 520 nm amplitudes for samples without and with TEA, respectively, demonstrating a clear difference in the growth of that absorption between the types of samples.

**Figure 8:**
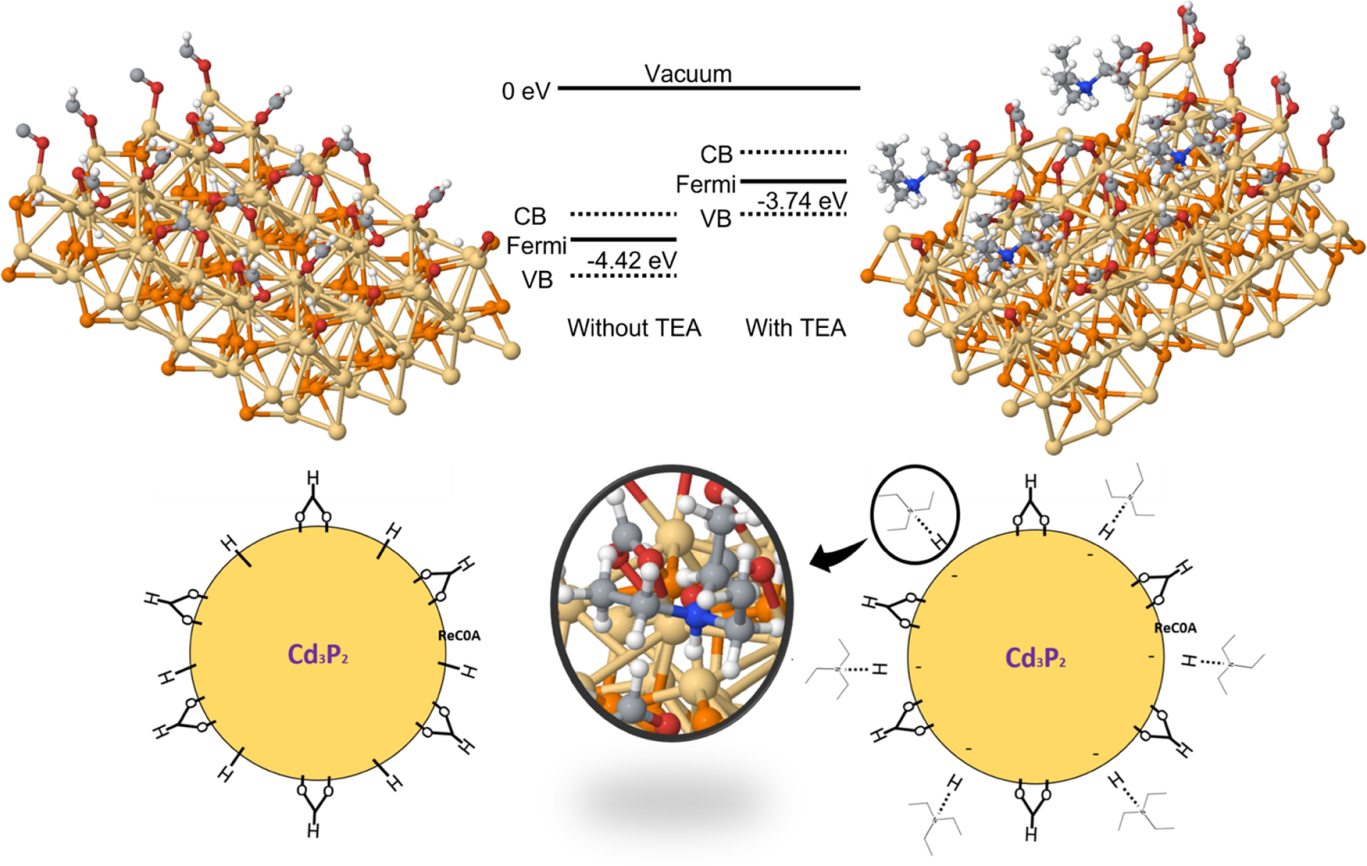
Shift of the Fermi level of Cd_3_P_2_ upon TEA addition to the surface. The addition of TEA abstracts protons from the Cd_3_P_2_ surface, resulting in a negatively charged surface, thus shifting up the Fermi level of the Cd_3_P_2_ quantum dot.

Many factors involving TEA may contribute to the observed increased in ET rate, including the following: TEA shifts the conduction band edges of QDs to a more negative potential by binding to the surface, donating electron density as it is an L-type ligand, as reported previously by Morgan et al.;^21^ the TEA deprotonates the QD surface since it is a strong, non-nucleophilic base; and TEA may preferentially solvate the QD resulting in a slightly different electrostatic environment, as suggested by the NMR experiments. Our computational modeling is more consistent with the picture that TEA deprotonates the QD surface, resulting in negatively charged QD surfaces, thus shifting the conduction band edge to more negative potentials (Figure 8). All of these factors can possibly change the energetics of the QD toward a more negative conduction band edge and favor faster single-electron transfer to the acceptor.

To attempt to confirm the shifting of the CB edge, we performed a TEA concentration-dependent study with Cd_3_P_2_–0.5× ReC0A, the smallest concentration used throughout our study. At 0.5× Re, upon addition of 0.5, 1, and 5% TEA, it is clear that the ET to ReC0A is accelerated (Figure S12). As more TEA is added, it may continue to shift the CB edge further as a result. The higher the concentration of TEA added, the more TEA there would be to possibly alter the QD surface charge by deprotonation, preferentially solvate the QD, and bind to the surface to donate electron density. This serves as more potential evidence that TEA may shift the CB edge to facilitate faster ET to ReC0A. The half-lives of QD–0.5xRe with TEA were plotted and show a linear relationship similar to the ReC0A concentration-dependent study.

For completeness, we have tested the effect of TEA addition on other electron transfer systems, including CdS–ReC0A, CdSe–ReC0A, CdS with methyl viologen, and Cd_3_P_2_ with 4,4′-bipyridine. Each of these systems has different surface structures due to their different chemical compositions as well as different capping ligands and various electron acceptors. Though the conditions for each case varied, a similar enhanced electron transfer rate was observed for all of those systems (Figure S19), indicating that the properties of TEA could be changing the conduction band edge through a combination of proton extraction, L-type ligand binding, and preferential solvation is generalizable to other systems, leading to enhanced electron transfer to electron acceptors in various systems beyond Cd_3_P_2_.

## Conclusions

We have shown that full consideration of the effects of incorporation of hole scavengers in catalytic systems must be considered. In particular, we have found through our combined computational and experimental analysis that the hole scavenger TEA may have the ability to change the conduction band potential of the QD through multiple possible mechanisms, including surface proton abstraction since it is a strong non-nucleophilic base. Binding of TEA to the surface and passivating surface traps is predicted to be less likely according to our computational modeling due to the steric hindrance. The possible favorable change in the conduction band potential enhances the rate of electron transfer reactions from Cd_3_P_2_, an effect that is generalizable to other systems (e.g., CdS–ReC0A, CdSe–ReC0A, CdS–methyl viologen, etc.). Preferential solvation of the nanoparticle complex may contribute to small changes in the immediately surrounding electrostatic environment. Aggregation of the electron acceptor and an excess of the hole scavenger are expected to lower the electron transfer barrier from the QD to the catalyst. These findings thus suggest that other hole scavengers might also have a double role as observed for TEA by not only supplying electrons to the nanoparticle–catalyst system but also potentially changing the energetics of the system, making it more favorable toward ligand reduction.

## Supporting Information Available

Experimental details as well as size determination of Cd_3_P_2_ QDs and corresponding TEM, steady-state UV–vis, TA and TRIR data from additional experiments as well as controls, wavelength-dependent kinetics of Cd_3_P_2_–ReC0A, time-resolved PL, ^1^H and DOSY NMR, CVs of ReC0A and ReCl with and without TEA and details of calculations performed (PDF)
