# Supplementary material for: Amine Hole Scavengers Facilitate Both Electron and Hole Transfer in a Nanocrystal/Molecular Hybrid Photocatalyst
Source: J Am Chem Soc. 2023 Jan 27;145(5):3238–47. doi: 10.1021/jacs.2c13464 (PMC9912264; doi:10.1021/jacs.2c13464)
Supplement: Supplementary file 1 — ja2c13464_si_001.pdf [file ja2c13464_si_001.pdf]

Supporting Information
Amine Hole Scavengers Facilitate both Electron and Hole Transfer in a Nanocrystal/Molecular Hybrid Photocatalyst

Sara T. Gebre[†o], Laura M. Kiefer[†o※], Facheng Guo[⁑], Ke R. Yang[⁑], Christopher Miller[‡], Yawei Liu[†], Clifford P. Kubiak[‡], Victor S. Batista[⁑], Tianquan Lian*[†]
† Department of Chemistry, Emory University, Atlanta, Georgia 30322, United States
‡ Department of Chemistry and Biochemistry, University of California, San Diego, 9500 Gilman Drive, MC 0358, La Jolla, California 92093, United States
⁑ Department of Chemistry, Yale University, New Haven, Connecticut 06511, United States
[o] *Authors contributed equally to this work*
※ *Currently at Department of Chemistry, Oakland University, Rochester, Michigan 48309, United States*

Contents

S1. Experimental

S2. Cd_3P_2 Size Determination

S3. HR TEM Experiments

S4. Attempting to Reverse Exciton Band Blue Shifting

S5. UV-Vis of ReC0A on CdS and CdSe

S6. TA Spectra of ReC0A + TEOA and QD + Methyl Viologen

S7. Transient IR Spectra of ReC0A on Cd_3P_2

S8. Loss of Bleach Amplitude

S9. TA Spectra of Cd_3P_2 and Different Concentrations of ReC0A on Cd_3P_2 with and without TEA

S10. TA Spectra of Cd_3P_2 + ReC0A with different concentrations of TEA

S11. Time-Correlated Single Photon Counting

S12. H NMR and DOSY of Cd_3P_2 and TEA

S13. Cyclic Voltammetry of ReC0A and TEA

S14. Kinetics of QDs and Electron Acceptors with and Without TEA

S15. *Ab initio* Method Details

S16. Quantum Dot Area Calculation

S17. References

S1. Experimental

Quantum Dot Synthesis. Cd_3P_2 QDs were synthesized according to the procedure described by Wu *et al.*[1] A mixture of CdO (77 mg), oleic acid (0.4 mL), and ODE (9 mL) was vented using a Schlenk line and alternated between being put under vacuum and argon for 1 min each, five times at approximately 75 °C. The temperature was then raised to 230 °C under Ar until the solution appeared clear. The temperature was raised once again to 250 °C and tris(trimethylsilyl)phosphine in ODE (0.2 mmol, 0.5 mL) was added, allowed to react for 35 sec, and removed from heat. The flask was put into a water bath to cool and stop the reaction from proceeding further. A small amount of toluene was added to the reaction mixture and transferred to 2 15 mL falcon tubes. Excess of ethanol was added to each falcon tube to help precipitate out the QD. The sample was centrifuged for 5 min at 5500 rpm three times, each time removing the supernatant and adding a small amount of toluene and a large volume of ethanol. The sample was dissolved in the solvent of choice, chloroform, and centrifuged one last time after which the supernatant was collected, and UV-Vis taken to determine the absorption of the QD. The diameter of the QDs are about 3 nm from TEM and the UV-vis absorption peak lies at 760 nm.

Sample Preparation. Previous methods of adsorbing molecules to QD surfaces involve adding an excess of the molecule and sonicating in a solvent (heptane) in which the quantum dot is soluble, but not the molecule. Instead, the samples were freshly prepared on the day experiments were performed by adding ReC0A in MeCN (2 mL) to a volume of Cd_3P_2 in chloroform (~0.33 mL, 75 μM) corresponding to a UV-Vis absorbance of 0.3 optical density (OD) for both in a 1:1 ratio of ReC0A (400 nm) to Cd_3P_2 (exciton, ~700 nm). The sample was rotary evaporated, dissolved in 1 mL of heptane to result in a final QD concentration of 25 μM, and sonicated for 3 hours to allow the ReC0A to bind to the QD. We added ReC0A to the QD solution and determined through FTIR spectral analysis that approximately 60% of the ReC0A added bound to the surface, resulting in a final concentration of 0.52 mM and an average of 21 Re complexes per QD. All samples were transferred into a 1 mm optical glass cuvette obtained from Starna and stirred for experiments. For experiments with the hole scavenger, 10% TEA was added to the already prepared Cd_3P_2-ReC0A samples in the dark and were wrapped in aluminum foil when transporting the cuvette. All chemicals were used as purchased and the ReC0A was synthesized using the following procedure by the Kubiak group at University of California San Diego. The TA experiments were compared with and without Ar purging and no difference was evident.

Visible Femtosecond Transient Absorption Spectroscopy. A Coherent Systems Ti:Sapphire regenerative amplifier system (800 nm fundamental, 2 W power output, 150 fs pulse width, 1 kHz repetition rate) with a Helios spectrometer (Ultrafast Systems LLC) was used for the visible picosecond experiments. This setup is further described in a related study by Wu *et al.*[1] The 800 nm fundamental laser output was split to generate the pump and probe beams. The fundamental beam was frequency doubled through a BBO crystal to get the 400 nm pump. The beam was

focused at the sample and a chopper was used, at a rate of 500 Hz. The second half of the 800 nm beam was focused onto a sapphire window to generate a white light continuum (WLC) as the probe. To get a ΔA spectrum, the WLC was split to form reference and signal beams and were focused into fiber coupled multichannel spectrometer with a complementary metal-oxide semiconductor (CMOS) for detection.

Transient Femtosecond Infrared Absorption Experiments. A Coherent Systems Astrella Ti:Sapphire regenerative amplifier system laser system was used (800 nm, 5 W power output, 35 fs pulse width, 1 kHz repetition rate) with infrared (IR) and visible optical parametric amplifiers (OPAs) and an Helios Fire transient absorption spectrometer (Ultrafast Systems LLC) to do transient infrared experiments. The 500 and 600 nm pump beams were generated from the visible OPA by sum frequency generation of the signal and by second harmonic generation of the signal through a BBO crystal, respectively. The pump beams were chopped at a rate of 500 Hz to obtain a ΔA spectrum and were also directed onto a motorized delay stage to get transient results. From the IR OPA, the signal and idler were mixed in an AgGaAs DFG crystal to form the 5000 nm IR probe**.** An iHR 320 Horiba spectrometer (component of Helios Fire setup) was used with a 50 grooves/mm grating. A nitrogen cooled 128 x 128 pixel mercury cadmium telluride (MCT) detector was used. Samples for the TRIR experiments were prepared in the same way as was done for visible transient experiments, described above.

Time Correlated Single Photon Counting (TCSPC). A mode-locked Ti:Sapphire laser (Tsunami oscillator pumped by a Spectra Physics 10 W Millenia Pro) was used with a fundamental output beam of 800 nm (~100fs, 80 MHz). The 800 nm beam traveled through a pulse picker (Conoptics, USA) and was then frequency doubled to generate the 400 nm excitation beam that would pass through the 1 cm cuvette containing the sample (right angle geometry). For detection, a microchannel plate photomultiplier tube (Hamamatsu R3809U-51) was used, and the analysis done by a TCSPC board (Becker & Hickel SPC 600). Similar to the transient absorption experiments, samples were prepared the day experiments were performed. With the QD and hole scavengers, Cd_3P_2 in chloroform was diluted 100x from the original amount used for TAS experiments (so that it had a UV-Vis absorbance of 0.1 OD in a 1 cm quartz cuvette) and 10% TEA was added (by volume). Samples were transferred to 1 cm cuvettes.

Electrochemistry and Cyclic Voltammetry (CV). Solvents were obtained from Fisher Scientific. Acetonitrile was degassed with argon, dried over alumina, and dispensed by a custom-made solvent system. Reagents obtained from commercial sources; pentacarbonylchlororhenium, decamethylferrocene (Fc*), and 2,2'-bipyridine from Sigma Aldrich, 2,2'-bipyridine-4,4'-dicarboxylic acid from Alfa Aesar, Tetrabutylammonium hexafluorophosphate ($TBAPF_6$, Aldrich, 98%) was recrystallized twice from methanol and dried at 90°C overnight. Triethylamine (Sigma ≥ 99%) was distilled from CaH_2 and stored over 3Å sieves in a N_2 glovebox prior to use. Experiments were performed on a BASi Epsilon potentiostat. Experiments were run in 0.1 M $TBAPF_6$ in 5 mL acetonitrile (MeCN) with 1 mM catalyst for control experiments. Experiments involving TEA were run in 0.1 M $TBAPF_6$ in 4.5 mL MeCN and 0.5 mL TEA with 1mM catalyst. Decamethyferrocene (1 mM) was used as an internal standard for all scans. The reported potentials were converted to NHE using literature values.[2-3] A 20 mL scintillation vial with a custom cap was used for all CV experiments. A 3 mm diameter glassy carbon working electrode, Pt wire counter electrode, and Ag/AgCl reference electrode (separated from solution in a glass tube filled with 0.1

M TBAPF$_6$ in MeCN and fitted with a CoralPor tip). The glassy carbon electrode was polished with 15, 3, and 1 micron diamond successively then thoroughly rinsed with methanol and dried before use. The platinum wire was flame-treated with a butane torch before use. Ar and "bone dry" CO_2 were run through Drierite columns and then through a sealed vial of dry MeCN filled with 3Å sieves. An oven dried cannula was used to transfer the MeCN saturated solution from the vial to the electrochemical set up. Electrochemical set-ups were sparged for at least 10 minutes prior to electrochemical experiments. Gas flow was continued over the solution during experiments. Ohmic drop was correct for by using the iR-compensation tool of the potentiostat. The tool corrected for between 90-100% of the measured resistance.

S2. Cd_3P_2 Size Determination

Using exciton band gap and TEM data from Wu et al, we fitted the band gap vs. size to the polynomial:

$$D = (0.00001258)\lambda_{BG}^2 - (0.01319)\lambda_{BG} + 5.806$$

where D is the nanoparticle diameter and λ_{BG} is band gap, both in nm.[1, 4]

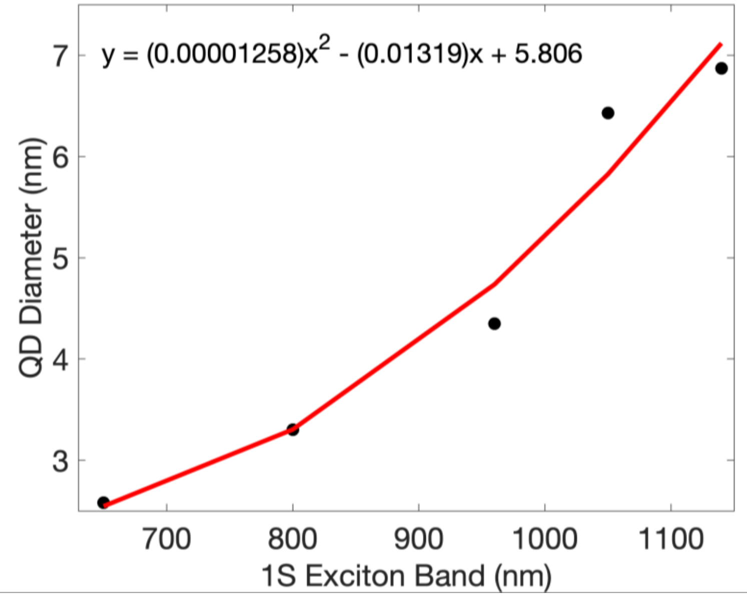

Figure S1. Polynomial of Cd_3P_2 band gap vs diameter.

S3. HR TEM Experiments

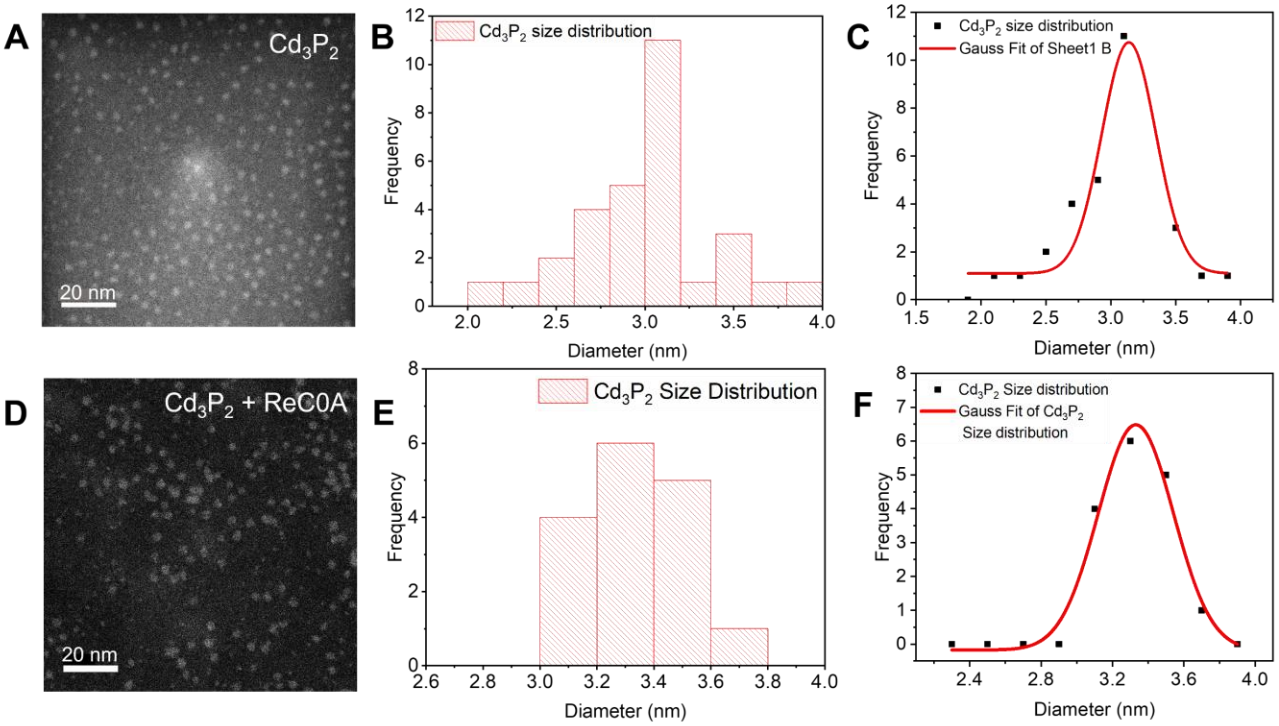

Figure S2. A) HR TEM image of Cd_3P_2, B) Histogram of size distribution of Cd_2P_3 QDs, C) Gaussian fit of histogram in (B) showing a size distribution of 3.14 ± 0.208 nm, C) HR TEM image of Cd_3P_2 with ReC0A on it, E) Histogram of size distribution of QDs in (D), F) Gaussian fit of histogram in (E) showing a size distribution of 3.33 ± 0.211 nm.

HR TEM experiments were conducted at Oakridge National Laboratory. Aberration-corrected high-angle annular dark-field (ADF) and bright-field (BF) image pairs were obtained on a Nion UltraSTEM U100 operated at 100kV. Samples were deposited on lacey carbon grids and baked at 80°C overnight under high vacuum to minimize hydrocarbon contamination. The average Cd_3P_2 diameter was determined to be 3.14 ± 0.208 nm (Figure S2A-C) and the Cd_3P_2-ReC0A had an average diameter of 3.33 ± 0.211 nm (Figure S2 D-F). Though these sizes aren't conclusive, it is clear from the images that the ReC0A did not etch the QD.

S4. Attempting to Reverse Exciton Band Blue Shifting

To reverse the blue shifting of the exciton band upon catalyst adsorption, 20% oleic acid (by volume) was added to the Cd_3P_2-ReC0A samples. Unfortunately, these results were inconclusive due to significant degradation of the QD. This experiment was repeated at two elevated temperatures, 57 and 108 ^{o}C, for both Cd_3P_2 and Cd_3P_2-ReC0A samples. After addition of ReC0A, there is a 20 nm blue shift that occurs and we found that this effect was not reversible upon addition of varying concentrations of OA (1 to 20%) and that there was a continual blue shift of up to 15 nm for both the QD and QD-ReC0A samples. We attribute the blue shift upon catalyst adsorption to strong electronic coupling of the catalyst and a change in the QD surface dipole as Cd_3P_2 is

strongly quantum confined and more sensitive to its surface environment. This is suggested by a continual blue shift with increasing amounts of Rec0A shown below and we will further investigate this at a later date.

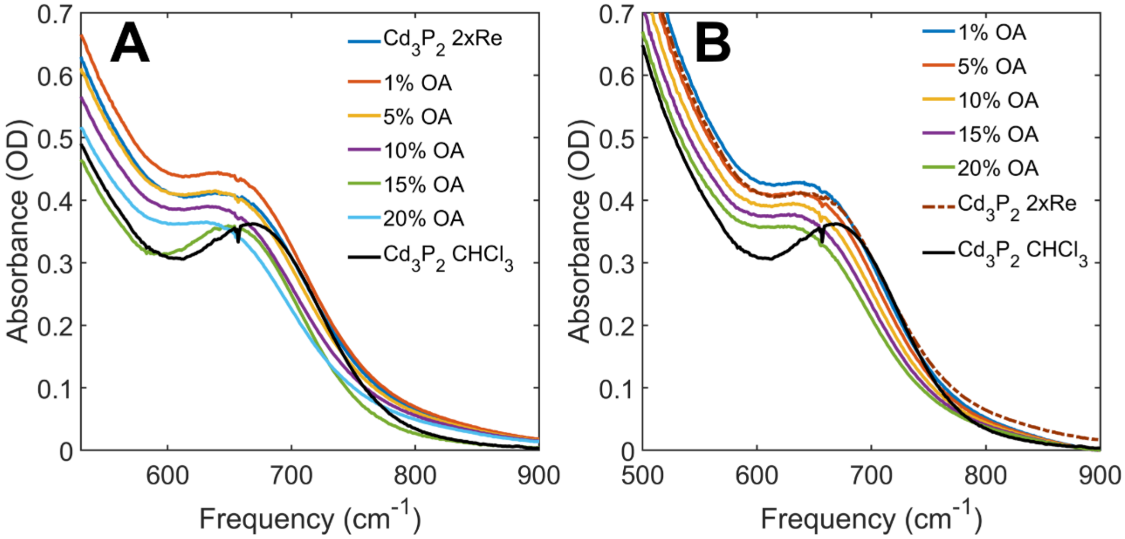

Figure S3. UV-Vis spectra after addition of oleic acid to Cd_3P_2-ReC0A samples at 57 (A) and 108 °C (B). Both spectra show that the exciton band further blue shifts upon addition of OA.

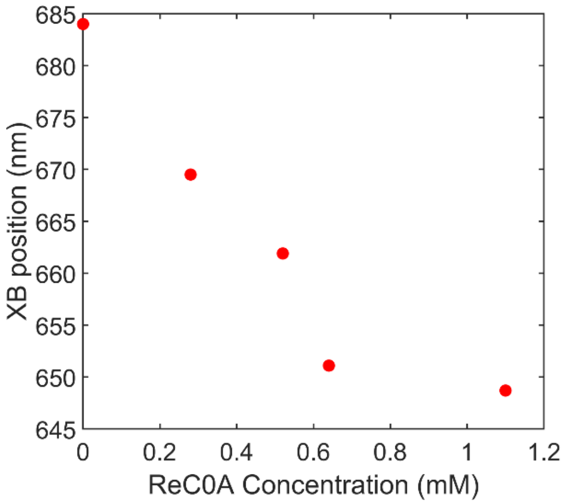

Figure S4. Plot of the exciton bleach shifting as a function of increasing ReC0A concentration. The more ReC0A bound to the surface of the QD, the farther the exciton band blue shifts.

S5. UV-Vis of ReC0A on CdS and CdSe

UV-Visible spectra for CdSe and CdSe-ReC0A in heptane and CdS and CdS-ReC0A in $CHCl_3$ and heptane, respectively. Note that there is no blue shift of the 1S exciton band upon addition of the ReC0A to either CdSe or CdS that was observed with Cd_3P_2. We attribute this to Cd_3P_2 being more quantum confined than the former QDs and more susceptible to surface dipole effects.

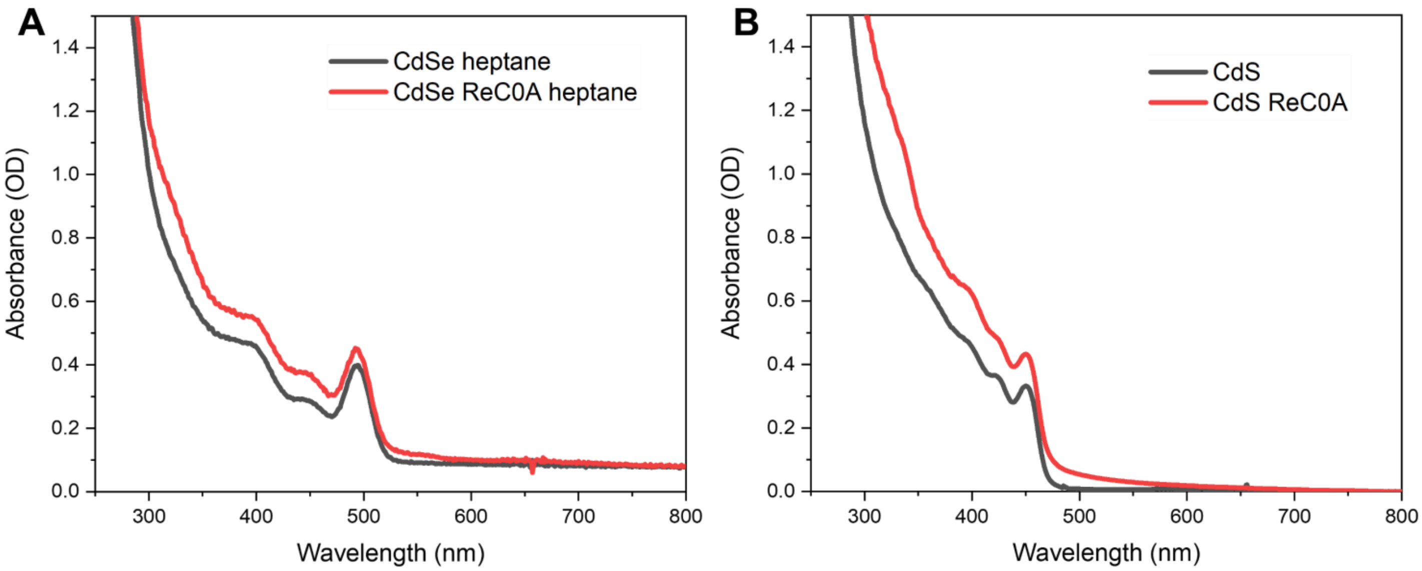

Figure S5. UV-Vis spectra of A) CdSe and CdSe-ReC0A (in heptane) and B) CdS (in $CHCl_3$) and CdS-ReC0A (in heptane).

S6. TA Spectra of ReC0A + TEOA and QD + Methyl Viologen

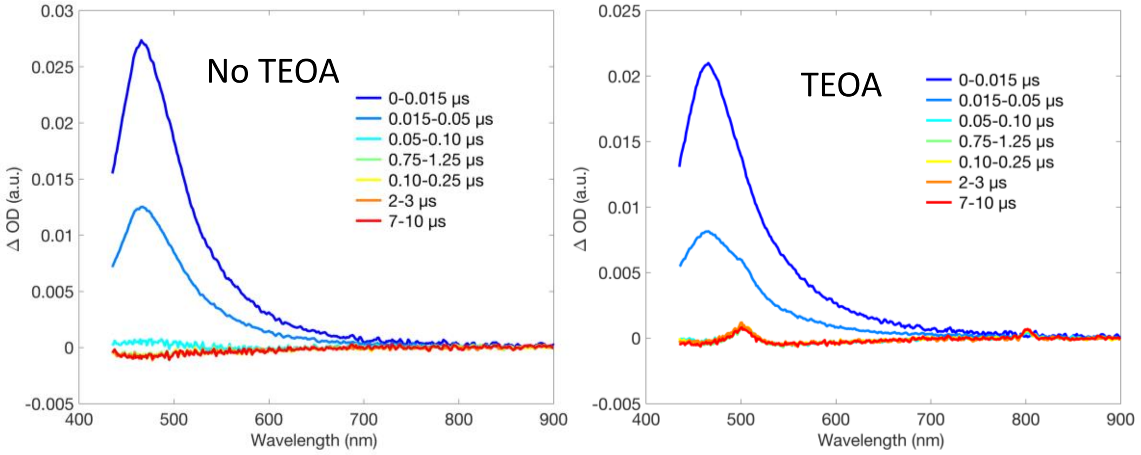

Figure S6. Nanosecond TA spectra of ReCl with and without TEOA. Upon excitation of the MLCT state at 400 nm, we observe the decay of the excited state (left). Once TEOA is added and the sample excited, the excited MLCT decays and at approximately 50-100 ns, a small absorption corresponding to the reduced ReCl species appears around 510 nm (right panel).

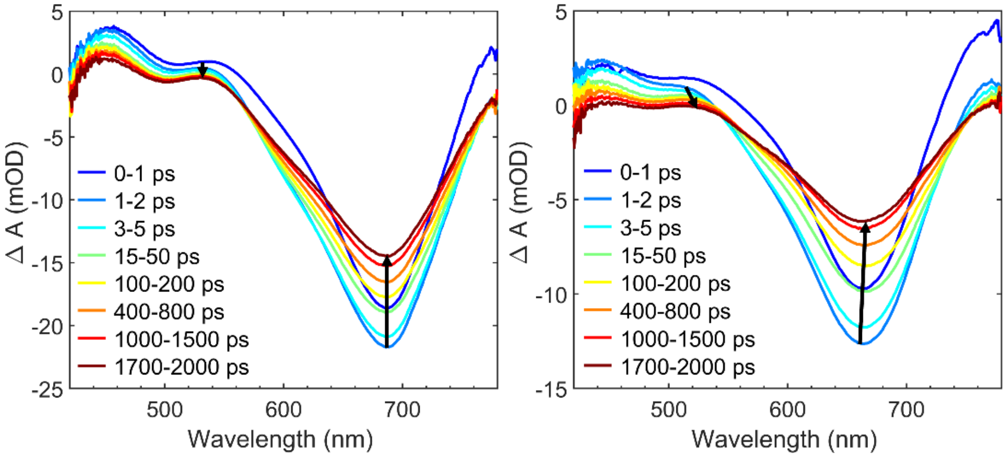

Figure S7. Cd_3P_2 without (left) and with (right) methyl viologen. After excitation with 400 nm pump of the QD-methyl viologen sample, there is a red shift of both the photoinduced absorption around 500 nm and the bleach at 680 nm. This is due to the growth of the charge separated state.

S7. Transient IR Spectra of ReC0A on Cd_3P_2

We performed transient IR (TRIR) experiments using 500 nm and 600 nm pump light, both outside of the ReC0A absorption range, and used ~ 2000 cm^{-1} centered broadband probe. The results show the ReC0A does indeed receive an electron from the Cd_3P_2, and at an ultrafast rate (Figure S7). The reduced Re species peak appears at a frequency ~20 cm^{-1} lower than that of the original species' bleach and within a 300 fs delay time. Others have also reported an ultrafast electron transfer, but have not speculated on the cause.[5]

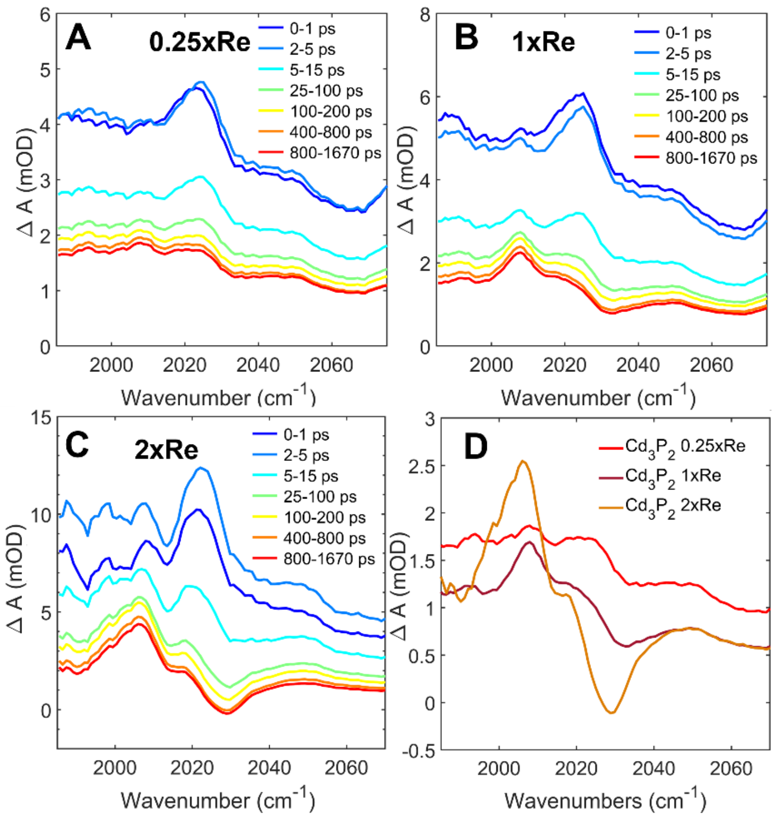

Figure S8. Transient IR spectra of ReC0A on Cd_3P_2 pumped with 500 nm at very high pump fluence showing that the Fano resonance signal forms almost immediately withing 0-1 ps. The offset is due to the excited electron signal in the quantum dot. A) Cd_3P_2-0.25xRe shows only a positive feature at 2025 cm^{-1} corresponding to the Fano resonance signal. B) Cd_3P_2-1xRe where Fano resonance can also be observed at early time, however, at ~2005 cm^{-1} there is a small absorption corresponding to the growth of the reduced ReC0A species. At later time, as the FR decays, that absorption becomes more pronounce and the ground state blaech of the complex is also observed at 2029 cm^{-1}. C) Cd_3P_2-2xRe. Similar features are observed here as part B, however, the decay of the FR is faster due to more ReC0A on the QD surface and there is also a larger reduced ReC0A signal corresponding to more reduced complex. D) Latest time delay (800-1670 ps) spectra overlayed for each sample after scaling 1x and 2xRe early time amplitudes to 0.25xRe data. As more ReC0A is added to the QD surface, there is more reduced ReC0A that appears faster.

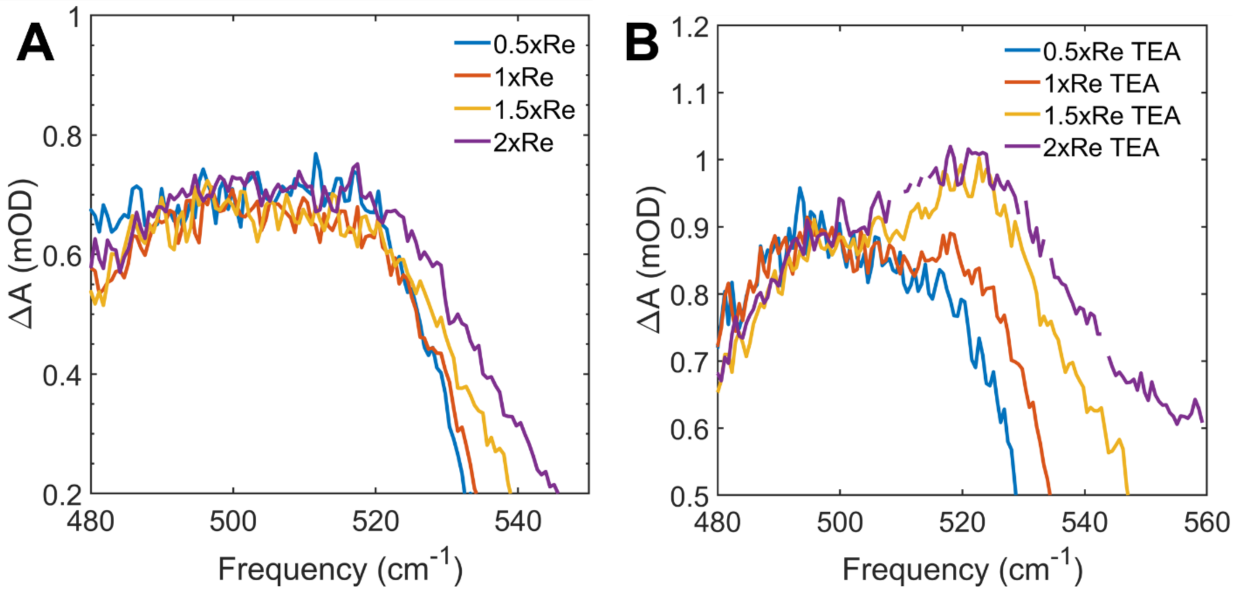

Figure S9. (A) and (B) show TA spectra at 1000-1670 ps for samples of each ReC0A concentration without and with TEA, respectively, zoomed into the 520 nm peak corresponding to reduced ReC0A seen in Figures 6C and 7C. Upon addition of TEA and faster bleach recovery, the reduced ReC0A signal becomes more visible.

S8. Loss of Bleach Amplitude

The loss of bleach amplitude we observe is most likely due to defect states as a result of OA likely being replaced upon ReC0A adsorption and ultrafast ET. With regards to the ultrafast ET, we can compare the unnormalized bleach amplitudes for Cd_3P_2 and Cd_3P_2-ReC0A at three different pump wavelengths: 400, 560, and 700 nm. Pumping at higher energy (400 and 560 nm) shows a bleach amplitude loss of almost half, while at 700 nm excitation, the bleach amplitude is the essentially the same for both samples. Both 400 and 560 nm pumps are at higher energy than the QD CB edge, so ultrafast ET is more likely to occur, whereas, at 700 nm, the same energy as the QD exciton band, there is most likely direct excitation to the CB edge. These results along

with our hypothesis that electron trapping due to ReC0A adsorption occurs likely both contribute to the loss in bleach amplitude.

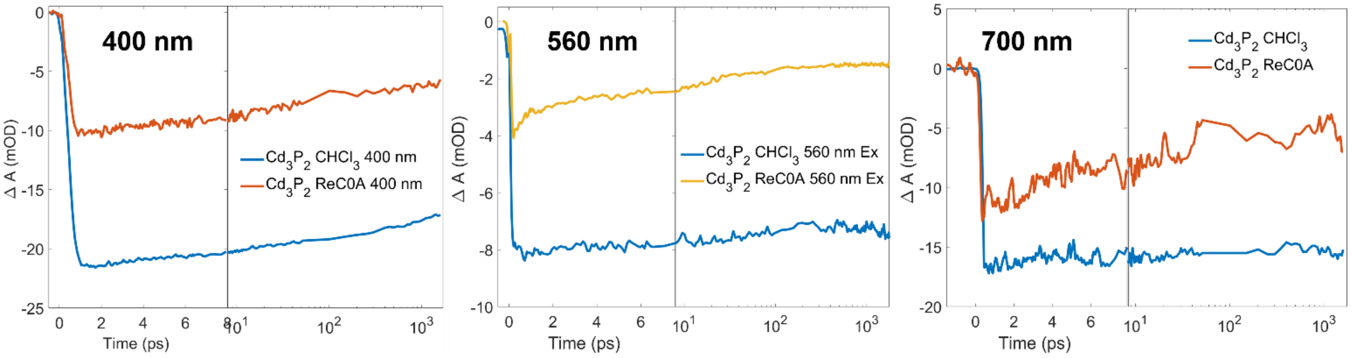

Figure S10. Unnormalized kinetics of Cd_3P_2 and Cd_3P_2-ReC0A at A) 400 nm, B) 560 nm, and C) 700 nm excitation. The higher energy pump wavelengths result in ultrafast electron transfer due to exciting much higher than the conduction band edge, while pumping at 700 nm likely results in direct band edge excitation.

S9. TA Spectra of Cd_3P_2 and Different Concentrations of ReC0A on Cd_3P_2 with and without TEA

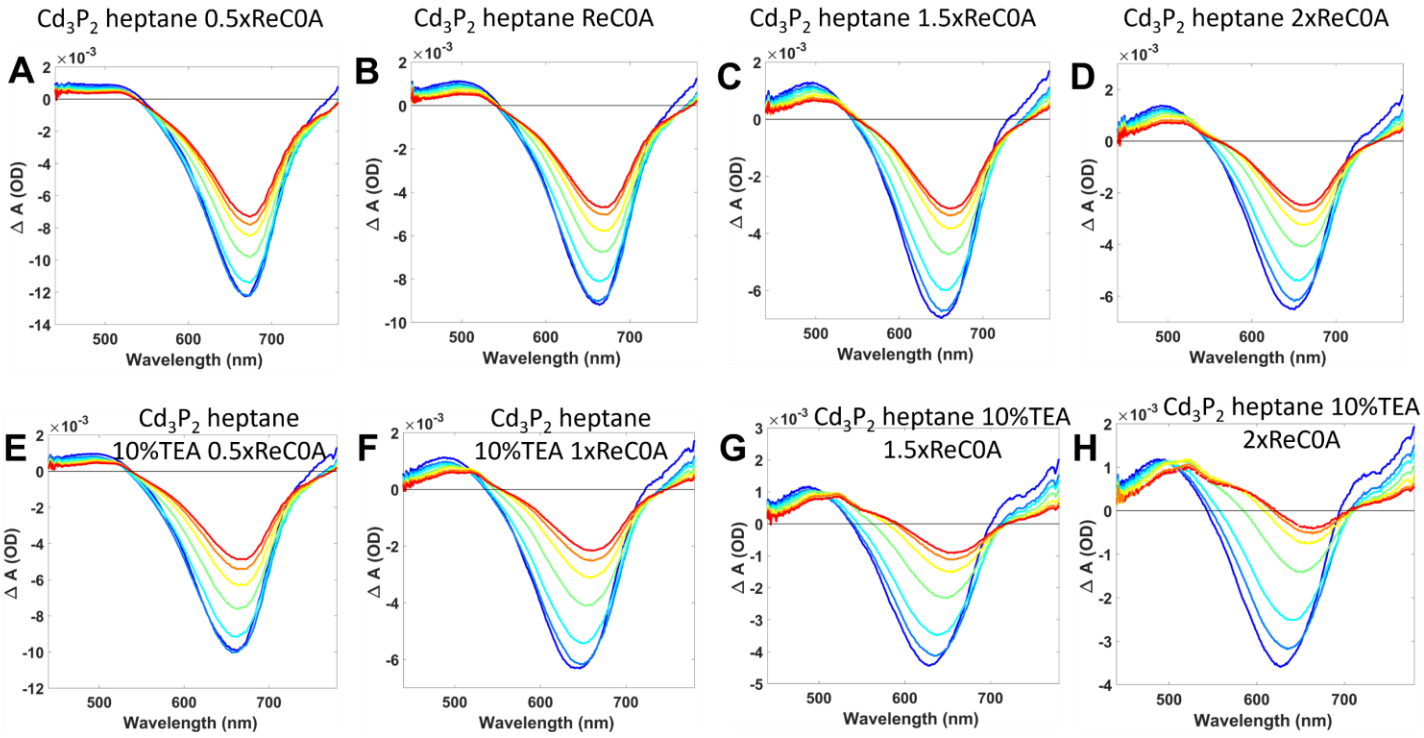

Figure S11. Transient absorption spectra of (A) 0.5xReC0A on Cd_3P_2, (B) 1xReC0A on Cd_3P_2, (C) 1.5xReC0A on Cd_3P_2, (D) 2xReC0A on Cd_3P_2 in heptane, and of (E) 0.5xReC0A on Cd_3P_2 with 10%TEA, (E) 1xReC0A on Cd_3P_2 with 10%TEA, (G) 1.5xReC0A on Cd_3P_2 with 10%TEA,

(H) 2xReC0A on Cd_3P_2 with 10%TEA all in heptane. [Legend: dark blue: 0-2 ps; blue: 2-5 ps; light blue: 5-15 ps; green: 25-75 ps; yellow: 100-300 ps; orange: 500-800 ps; red: 1000-1670 ps;]

The top four panels of Figure S6 show the evolution of TA spectra over time for the various concentrations of ReC0A on the Cd_3P_2 in heptane. As more ReC0A is added, the band blue shifts. While a blue shift often occurs from degradation of the QD, our TEM data shows that degradation is not occurring, as the size is slightly larger with the ReC0A. The bottom four panels show the same exciton bands after addition of 10% TEA. They show an even further blue shift. In both sets of panels, the 1.5x and 2x ReC0A concentrations show a clear charge separated state emerging over time with a derivative shape, the positive feature approximately between 400 nm and 650 nm and a negative feature between 650 nm and 720 nm.

S10. TA Spectra of Cd_3P_2 + ReC0A with different concentrations of TEA

We performed TA experiments of Cd_3P_2-0.5xReC0A with varying concentrations of TEA to observe the effect on ET. We found that as you increase TEA concentration, ET also increases, shown in the figure below.

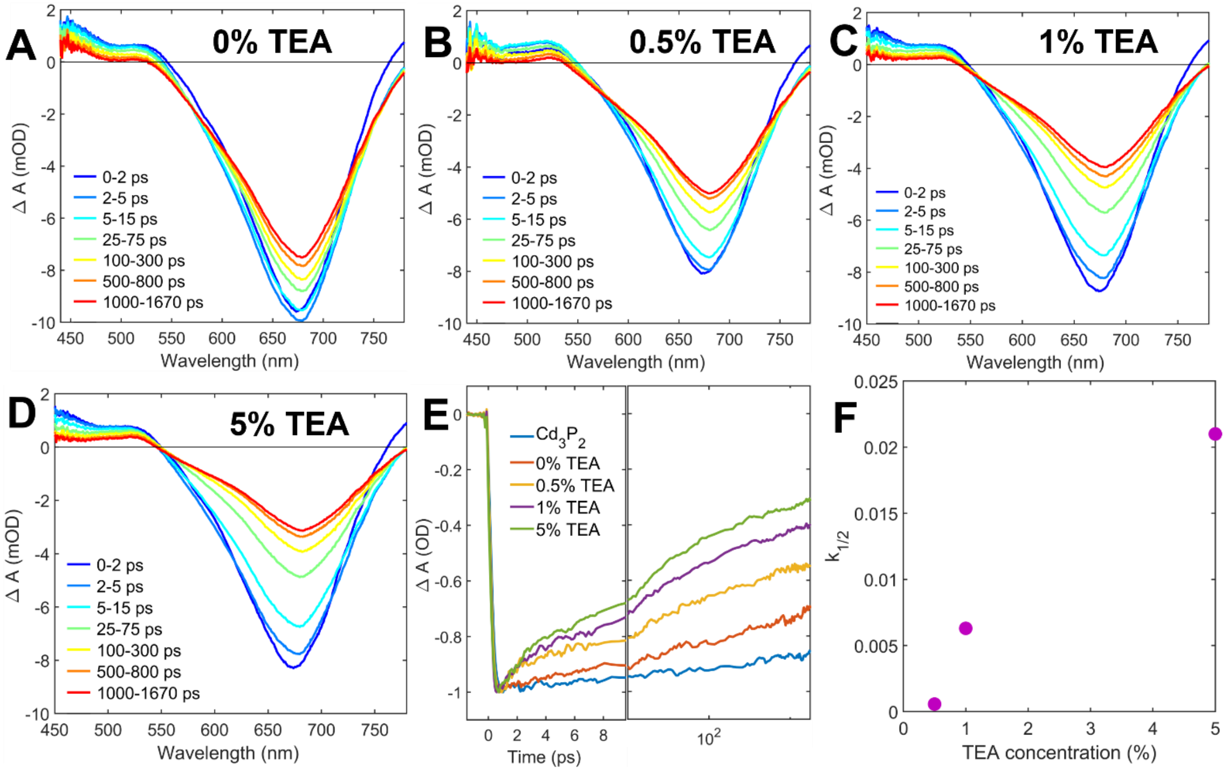

Figure S12. TA spectra with varying concentrations of TEA (by volume) with constant ReC0A concentration held at 0.5xRe (0.28mM) bound to Cd_3P_2. A) 0% TEA (Cd_3P_2-0.5xReC0A), B) 0.5% TEA, C) 1% TEA, D) 5% TEA, E) kinetics for each of these samples including Cd_3P_2 only, and F) plot of half-life with increasing TEA concentration.

S11. Time-Correlated Single Photon Counting

The absorption and fluorescence spectra were taken of the Cd_3P_2 (Figure S7A). The fluorescence spectrum was used to determine a suitable wavelength for collection for time-correlated single photon counting experiments, which were conducted using 400 nm to excite and detected at 780 nm. Time-correlated single photon counting experiments were performed with various concentrations of TEA, showing that at less than or equal to 17.7 mM, the TEA slightly enhanced the fluorescence (Figure 7B), and at concentrations that were higher, the TEA behaved as a hole scavenger (Figure S7C), quenching the photoluminescence.

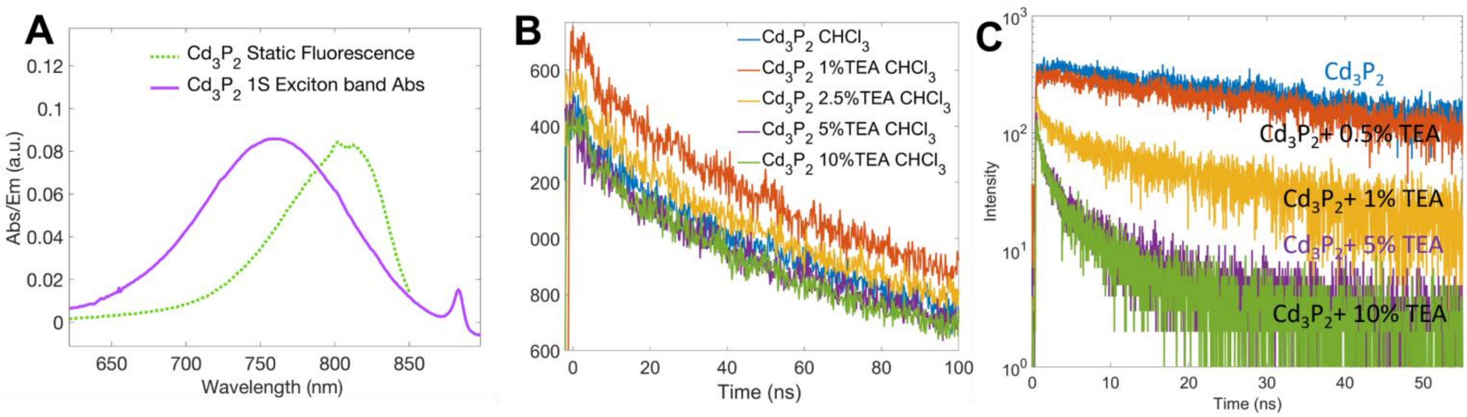

Figure S13. A) UV-Vis and Fluorescence of Cd_3P_2 in $CHCl_3$, B) TCSPC of Cd_3P_2 with varying low concentrations of TEA (in order: 7.1 mM, 17.7 mM, 35.5 mM, and 70.9 mM) and C) TCSPC of Cd_3P_2 with varying high concentrations of TEA.

S12. ^{1}H NMR of Cd_3P_2 and TEA

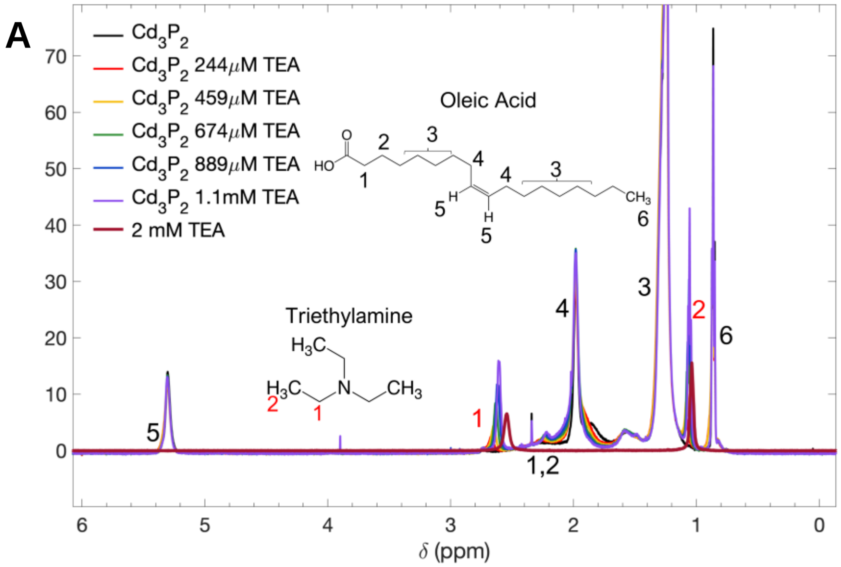

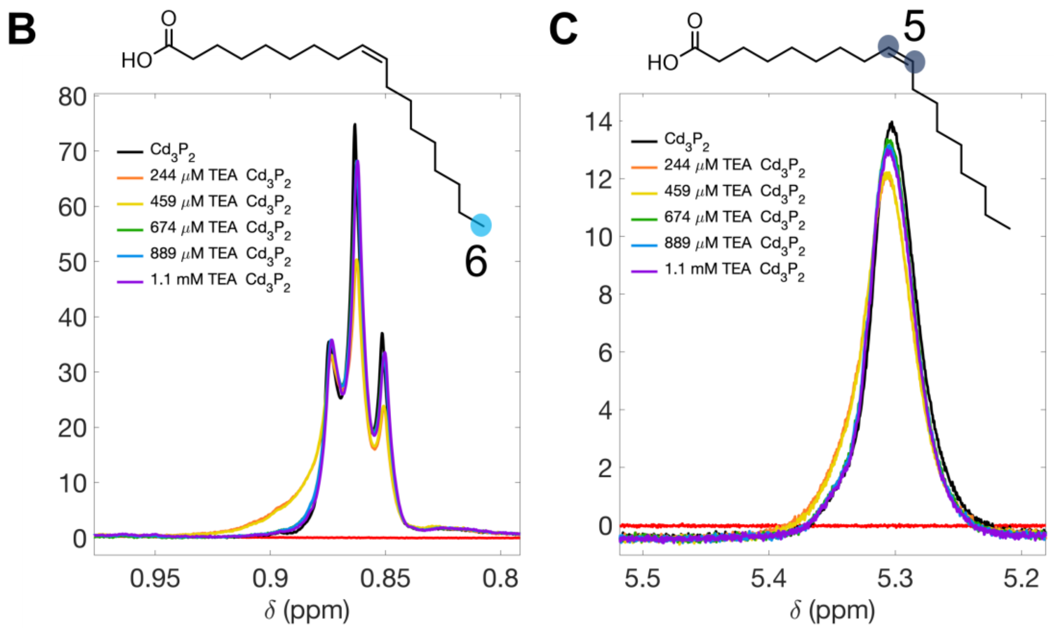

Figure S14. A) NMR spectra of Cd_3P_2 in $CDCl_3$ with varying concentrations of TEA B) CH_3 Oleic acid peaks and C) CH_2 Oleic acid peaks show broadening with addition of low concentrations of TEA and return to original peak width with higher concentrations.

Samples were prepared by rotary evaporating the Cd_3P_2, chloroform, and redissolving in $CDCl_3$ obtained from Sigma Aldrich (99.96% D atom). The concentration of the QD was calculated to be ~ 25 µM and varying concentrations, from 259 µM to 1.1 mM, of TEA in $CDCl_3$ were added to the sample for the titration. For ^{1}H NMR studies, a Bruker Ascend 600 MHz spectrometer was used (frequency: 600.18 MHz). It also included a prodigy cryoprobe that was cooled with liquid nitrogen. Figure S8 shows H NMR spectra of Cd_3P_2 in $CDCl_3$ with various amounts of TEA added.

DOSY NMR was performed on the same instrument described above with a TEA concentration of 459 µM (second lowest concentration). A gradient was applied increasing from 2 to 95% with 25 points taken. These points were then fit to the following equation to obtain the diffusion coefficient:

$$I = I_0 e^{-(\gamma g \delta)^2 * D(\Delta - \frac{\delta}{3})} \qquad \text{Eq. 1}$$

where I is the intensity, I_0 is the intensity at zero gradient strength, γ is the gyromagnetic ratio, g is the gradient strength, δ and Δ are the delays between pulses, and D is the diffusion coefficient.[6]

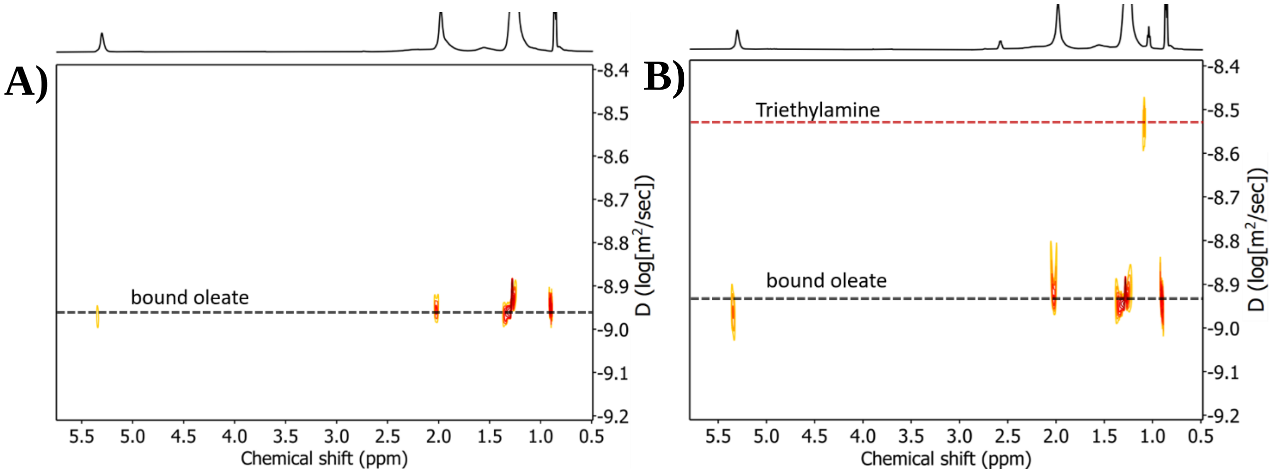

Figure S15. DOSY NMR spectra of a) Cd_3P_2 and b) Cd_3P_2 with TEA.

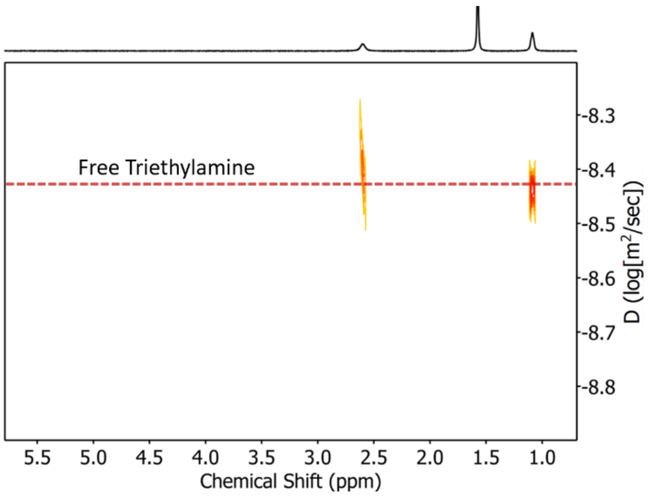

Figure S16. DOSY NMR spectra of TEA in $CDCl_3$ provides a diffusion coefficient of 3.62 $\mu m^2/s$.

S13. Cyclic Voltammetry of ReC0A and TEA

CV experiments were conducted on $Re(bpy)(CO)_3Cl$ (ReCl) in MeCN as a control. The first reduction of ReCl remains the same (-1.16 vs NHE) regardless of TEA's presence in solution and there is no appearance of a peak between the first and second reduction of the complex.

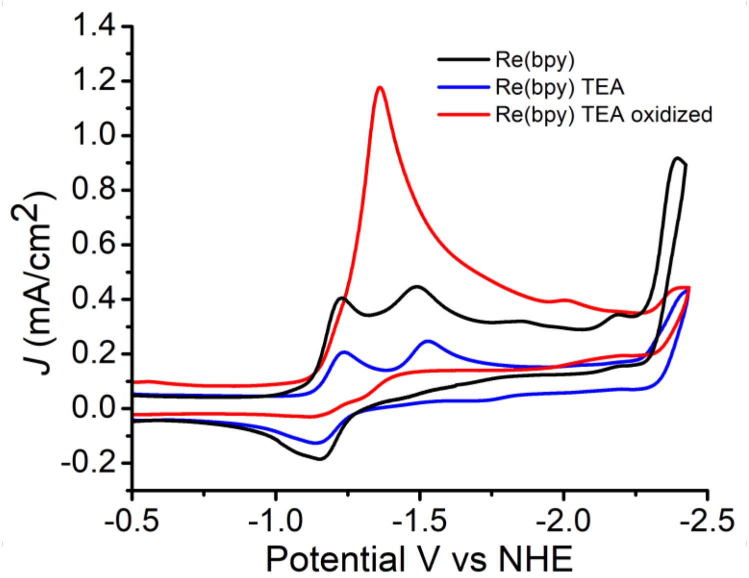

Figure S17. Cyclic voltammogram of Re(bpy)$(CO)_3$Cl with and without TEA.

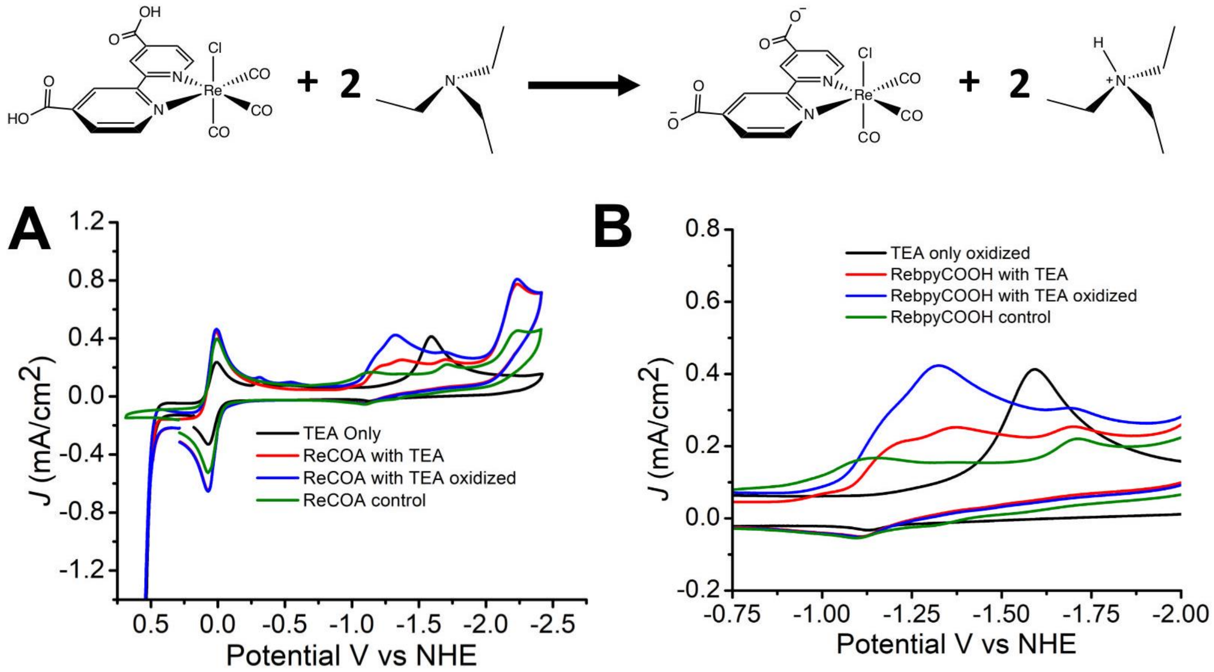

Figure S18. (A) CV curves for TEA, ReC0A, ReC0A + TEA and ReC0A + TEA with TEA oxidized first, (B) zoomed in region of the curves in (A).

To determine if the small peak in the ReC0A electrochemistry was from the reduction of $TEAH^+$, TEA oxidation and reduction was studied electrochemically. In a solution with TEA and no catalyst, there were not visible reduction peaks when scanning between 0.4 V and -2.0 V vs NHE. When the solution is scanned past 0.5 V vs NHE, there is a large increase in current corresponding to the oxidation of TEA. In the same scan, the reduction of $TEAH^+$ is now present at -1.56 V vs NHE. In the presence of ReCl, the oxidation of TEA is unchanged but, when the oxidation is

followed by scanning cathodically, the reduction of TEA shifts positively to -1.34 V vs NHE. When this experiment is run in the presence of ReC0A, the reduction of the $TEAH^+$ appears at -1.29 V vs NHE, similar to the small peak seen in the TEA plus ReC0A solution. From this, we conclude that the change in the first reduction of the ReC0A upon addition of TEA and the appearance of a new reduction peak, is due to the deprotonation of the carboxylic acid on the bpy ligand.

S14. Kinetics of QDs and Electron Acceptors with and Without TEA

To study if the increased electron transfer rate after adding TEA was generalizable, we tested multiple QD-electron acceptor systems with and without TEA, in multiple solvents. Figure S10, shows that this is indeed the case. We studied CdSe in heptane with ReC0A bound, CdS in $CHCl_3$ with ReC0A bound and CdS in $CHCl_3$ with methyl viologen ($[MV]^{2+}$) in solution. Each of them shows an enhanced electron transfer rate upon the addition of 10% (v/v) TEA. All samples were excited with 400 nm light.

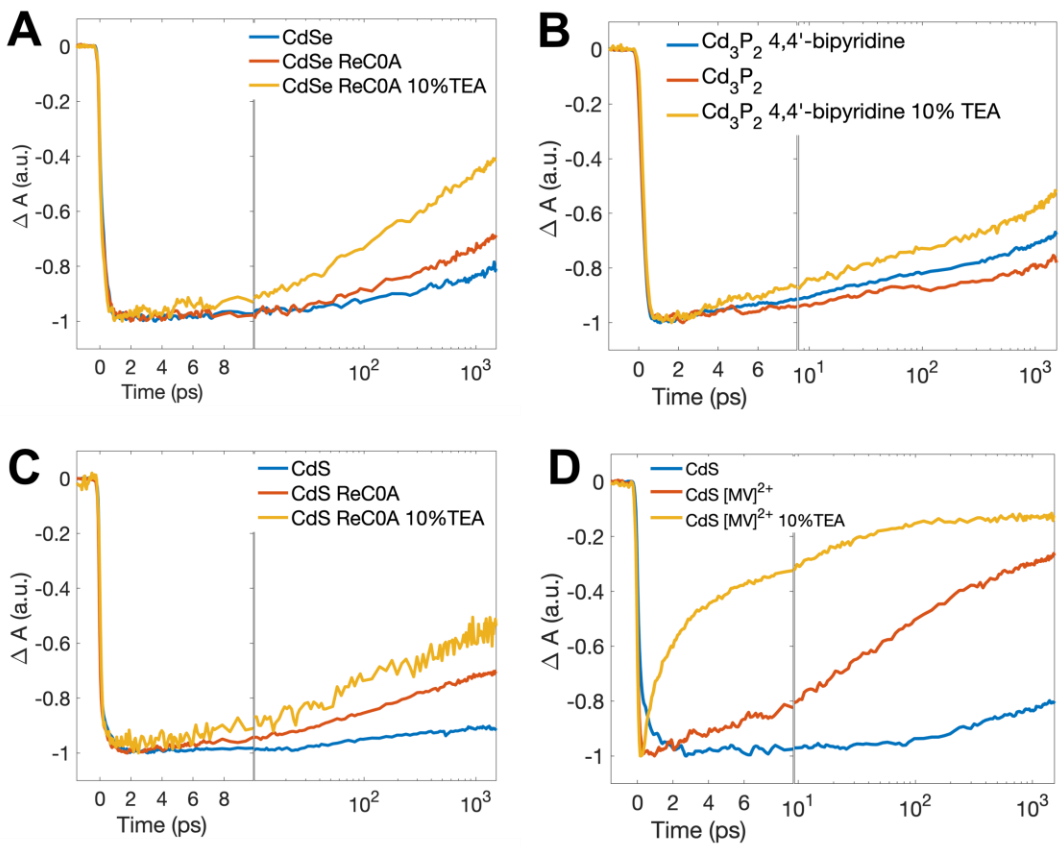

Figure S19. Normalized kinetic traces of different quantum dots, ReC0A bound, and TEA added, showing increased electron transfer with the addition of the hole scavenger A) CdSe, CdSe-ReC0A, CdSe-ReC0A-TEA B) Cd_3P_2, Cd_3P_2-4,4'-bipyridine, Cd_3P_2-4,4'-bipyridine-TEA C) CdS, CdS-ReC0A, CdS-ReC0A-TEA D) CdS, CdS-MV^{2+}, CdS-MV^{2+}-TEA.

S15. *Ab initio* Method Details

Gaussian 16 version A.03[7] and Vienna Ab initio Simulation Packages (VASP) version 5.4.1[8-11] were used to on all density functional theory (DFT) calculations. The Vaspkit[12] program was used to calculate the work function and Jmol[13] was used for visualization.

Gaussian was used for ReC0A geometry optimizations and frequency calculations, which were performed using the (U)B3LYP functional[14-16] with the 6-311+G(2df,p) basis sets[17-25] on all non-metallic atoms and the def2-TZVP effective core potential and basis set[26-27] on the Re atom. for geometry optimization and frequency calculations. Scale factor 0.965 was applied to all frequency calculation results reported in this article.

VASP was used for optimizations of Cd_3P_2 unit cell, Cd_3P_2 slab, Cd_3P_2 slab with capping agent, and Cd_3P_2 slab with capping agent & ReC0A. The electron–ion interactions were described by the Perdew–Burke–Ernerhof (PBE) exchange-correlation functional[28] and the projected augmented-wave (PAW) method[29-30]. The Gaussian smearing method with a smearing parameter $\sigma = 0.1$ eV was applied for all the calculations. The DFT-D3 method with the Becke-Jonson damping[31-32] was used to describe dispersion interactions. The plane wave basis set was cutoff at 400 eV and the energy convergence criterion was set to be 10^{-6} eV per unit cell. A Monckhorst–Pack[33] (MP) type k-point grid of 9 × 9 × 9 was used for the optimization of Cd_3P_2 unit cell; an MP type k-point grid of 3 × 3 × 1 was used for the optimization of Cd_3P_2 slabs (with and without capping agents); an MP type k-point grid of 1 × 1 × 1 was used for the optimization of Cd_3P_2 slab with capping agents and ReC0A. The capping agents were modeled as formic acid. During structure optimization, the Cd_3P_2 unit cell's atom position, cell shape and cell volume were allowed to relax; for Cd_3P_2 slabs, Cd_3P_2 slabs with capping agents, and Cd_3P_2 slabs with capping agent & ReC0A only atom positions were allowed to relax. However, atoms in the bottom half of Cd_3P_2 slabs were frozen at their bulk positions. Both ReC0A binding geometries were optimized with the same unit cell and number of molecules. For the single carboxylic acid case, the ReC0A was optimized from a monodentate structure. In the case of dicarboxylic acid binding, the complex was optimized from a bidentate structure. The stable (100) facet of Cd_3P_2 was selected as the exposed surface to model Cd_3P_2 quantum dots. For all Density of State (DOS) calculation, the Heyd-Scuseria-Ernzerhof (HSE) screened hybrid functional[34] was applied. Considering the size of the model, a MP type k-point grid of 1 × 1 × 1 was applied.

S16. Quantum Dot Area Calculation

In experiments, the QD diameter is 3.1 nm and the QD concentration is 25 μM. From the simulation model, we estimated for both ReC0A's carboxyl functional group to be bound on the QD surface, the minimum surface area require is 1.06 nm^2 per ReC0A molecule. The experimental QD's surface area is 30.19 nm^2, thus about 28.5 (31.19 nm^2/1.06 nm^2) ReC0A molecules can be adsorbed on the QD through both carboxyl groups. If the number of ReC0A molecules is more than 28.5 per QD, some of them can only have single carboxyl bound to the surface, with the second carboxyl group protonated and pointed away from QD. For a solution with 25 μM QD, the critical concentration for ReC0A to change its surface binding mode from double carboxylate to single carboxylate will be 0.71 (28.48*25 μM* 1/1000 mM/μM) mM, which is within the same range of experimental concentrations to see the disappearance of the 1700 cm^{-1} peak. The maximum effective concentration of surface protons should be 2.84 mM (4 times site number compared to ReC0A binding site)

S17. References

1. Wu, K. F.; Liu, Z.; Zhu, H. M.; Lian, T. Q., Exciton Annihilation and Dissociation Dynamics in Group II-V Cd3P2 Quantum Dots. *Journal of Physical Chemistry A* **2013,** *117* (29), 6362-6372.
2. Connelly, N. G.; Geiger, W. E., Chemical Redox Agents for Organometallic Chemistry. *Chemical Reviews* **1996,** *96* (2), 877-910.
3. Pavlishchuk, V. V.; Addison, A. W., Conversion constants for redox potentials measured versus different reference electrodes in acetonitrile solutions at 25°C. *Inorganica Chimica Acta* **2000,** *298* (1), 97-102.
4. Yu, W. W.; Qu, L. H.; Guo, W. Z.; Peng, X. G., Experimental Determination of the Extinction Coefficient of CdTe, CdSe, and CdS Nanocrystals. *Chemistry of Materials* **2003,** *15* (14), 2854-2860.
5. Huang, J.; Gatty, M. G.; Xu, B.; Pati, P. B.; Etman, A. S.; Tian, L.; Sun, J. L.; Hammarstrom, L.; Tian, H. N., Covalently Linking $CuInS_2$ Quantum Dots with a Re Catalyst by Click Reaction for Photocatalytic CO_2 Reduction. *Dalton Transactions* **2018,** *47* (31), 10775-10783.
